# Time-resolved transcriptomic profiling of mammary gland tissue during ductal morphogenesis, lactation activation, and involution in sows

**DOI:** 10.5713/ab.250560

**Published:** 2025-11-14

**Authors:** Yanan Peng, Biqing Xuan, Jinhao Tian, Yiyi Guo, Jinlin Cao, Linfan Zhang, Rong Xuan

**Affiliations:** 1 Key Laboratory of Livestock and Forage Resources Utilization around Tarim, Ministry of Agriculture and Rural Affairs, College of Animal Science and Technology, Tarim University, Alar, China; 2 College of Animal Science and Technology, Shandong Agricultural University, Tai’an, China

**Keywords:** Lactation Cycle, Mammary Gland Involution, Porcine, RNA-seq, Weighted Gene Co-expression Network Analysis

## Abstract

**Objective:**

Mammary development and lactation are vital for piglet survival, but gene expression profiles from gestation to early involution (W2) in sows remain unclear. This study profiles key transcriptomic changes to reveal molecular features.

**Methods:**

Mammary gland tissue samples were collected from hybrid half-sibling sows (Danish Landrace×Yorkshire) at five physiological stages: mid-gestation (MG), late gestation (LG), early lactation (EL), peak lactation (PL), and W2 (day 2 after weaning). Transcriptome sequencing (RNA-seq) was performed on 30 samples (n = 6 per stage). Differential expression analysis and clustering were conducted to identify expression patterns. Functional enrichment, pathway analysis, and weighted gene co-expression network analysis (WGCNA) were used to identify stage-specific regulatory networks and hub genes involved in mammary gland development, metabolism, immune response, and structural remodeling.

**Results:**

Transcriptome profiling yielded over 61,000 expressed transcripts, with 27,244 shared across all stages. A total of 12,239 transcripts were differentially expressed, with the greatest transcriptomic shift occurring between PL and W2 (4,829 differentially expressed transcript [DETs]). DETs were grouped into five expression clusters, each showing stage-specific enrichment in biological processes. W2-associated transcripts were enriched in pathways related to cell junction integrity and apoptosis, while MG and LG stages were associated with proliferation and metabolic pathways. EL and PL stages showed enrichment in immune and lipid metabolism pathways. WGCNA identified nine gene modules, with modules linked to gestational growth (brown, blue), lactation (green, turquoise), and involution (yellow, turquoise). Key regulatory genes such as *EGF*, *AKT1*, *SRC*, *GATA3*, *STAT6*, *TNFSF11*, and *NFKB1* were identified as central hubs within six major functional networks.

**Conclusion:**

This study constructed a time-resolved transcriptomic atlas of porcine mammary gland development, lactation, and involution. It reveals gene expression dynamics, identifies candidate pathways, and delineates molecular signatures associated with structural and functional changes in the mammary gland. The findings offer potential targets and a theoretical framework for improving sow lactation performance and regulating mammary function.

## INTRODUCTION

The mammary gland is essential for providing nutrition and immune protection to neonates, with its development, lactation, and involution regulated by dynamic molecular networks [1]. During gestation, it undergoes ductal and alveolar development, followed by secretory activation during lactation, and rapid remodeling after weaning [2]. In pigs, mammary function directly affects piglet survival and growth [3]. Therefore, characterizing transcriptomic changes and associated pathways in the porcine mammary gland from gestation to early involution (W2) can provide insights into the molecular mechanisms underlying functional transitions during the lactation cycle.

Several studies have contributed to our understanding of transcriptional changes during porcine mammary development and lactation. Fan et al [4] used scRNA-seq to profile mammary cell populations from late gestation (LG) to involution, while Keel et al [5] analyzed colostrum and mature milk transcriptomes across parities. Palombo et al [6] identified key genes regulating the transition from colostrogenesis to lactogenesis, and Zhao et al [7] characterized transcriptomic dynamics at gestational days 80, 100, and 110 [7]. Other studies examined hormonal regulation [8] and gene expression related to breast cancer traits [9]. Collectively, these works have provided important insights into transcriptional regulation at specific stages or under defined conditions. Nevertheless, many of these studies were conducted with limited sample sizes and concentrated on particular developmental windows or individual aspects of mammary function. A comprehensive, stage-by-stage analysis covering the entire cycle of development, lactation, and involution is currently absent.

In this study, we performed high-resolution RNA-seq across five key stages—mid-gestation (MG), LG, early lactation (EL), peak lactation (PL), and W2 (Day 2 post-weaning)—capturing the continuum of ductal expansion, alveolar formation, lactation initiation and maintenance, and subsequent involution. We constructed a dynamic transcriptional atlas of the sow mammary gland and, through differential expression, functional enrichment, and co-expression network analyses, identified key biological processes and regulatory factors at each stage. These findings offer novel insights into the molecular basis of mammary development and function, with implications for improving lactational performance and genetic selection in pigs.

## MATERIALS AND METHODS

### Animal handling and mammary tissue collection

Mammary gland tissue samples were collected from 30 multiparous hybrid half-sibling sows (Danish Landrace×Yorkshire, Supplement 2). All sows were individually housed in standardized farrowing crates at a large-scale pig farm affiliated with the Cooperative of Regiment 13, Aral City, Xinjiang Uygur Autonomous Region, to ensure environmental consistency and controllable sow health status. According to key physiological stages of mammary gland development and the lactation cycle, tissue samples were collected at the following five time points: day 70 of gestation (MG, characterized by continuous ductal development), day 110 of gestation (LG, characterized by progressive alveolar formation), day 2 postpartum (EL, characterized by rapid activation of secretory function), day 10 postpartum (PL, characterized by sustained milk secretion), and day 2 after weaning (W2, characterized by the onset of mammary gland remodeling). At each time point, six sows were selected as biological replicates. Prior to slaughter, sows were stunned using electrical stunning, followed by exsanguination. Immediate dissection was performed to expose the mammary glands. A standardized sampling protocol was followed: approximately 0.5–1 cm^3^ of parenchymal mammary tissue was excised from the left third mammary gland, located 1–2 cm below the teat, carefully avoiding contamination with fat, skin, and connective tissue. The collected tissue samples were rapidly frozen in liquid nitrogen within 15 seconds of excision and subsequently stored at −80°C for total RNA extraction and transcriptome sequencing. All sampling instruments were treated with RNase inhibitors prior to use to prevent RNA degradation.

### Mammary tissue sequencing and data analysis

Total RNA was extracted from porcine mammary gland samples using RNAiso Plus (Takara). RNA quality was assessed with a NanoDrop 2000C spectrophotometer, and samples with RNA integrity number (RIN)>8 were used for library construction. Thirty libraries were prepared using the TruSeq RNA Library Preparation Kit ver. 2 (Illumina) and sequenced on the Illumina HiSeq 2500 platform. Raw data have been deposited in the NCBI GEO database (accession number: GSE295679).

Sequencing quality was evaluated using FastQC (ver. 0.12.0), and reads were trimmed with Trimmomatic (ver. 0.40) to remove adapters, primers, poly-A tails, and low-quality bases. Clean reads were aligned to the *Sus scrofa* reference genome (GCF_000003025.6) using HISAT2 (ver. 2.2.1). SAM files were converted and sorted into BAM format using SAMtools (ver. 1.21). Transcript assembly and quantification were performed with StringTie (ver. 3.0.0). Transcript quantification and subsequent analyses were performed at the transcript level to capture the dynamics of comprehensive transcript expression. Each transcript was designated by its unique RefSeq accession number, and transcript isoforms with the same gene symbol were retained to preserve isoform-level resolution instead of being combined into a single gene-level value.

Transcripts with FPKM≥0.5 in at least one stage were retained. Normalization and correction of expression data for unwanted variation, primarily inter-individual differences unrelated to the time-series design, were performed using the RUVs function from the RUVSeq package (ver. 1.42.0). Principal component analysis (PCA) and relative log expression (RLE) analysis were conducted with the same package.

### Differential expression analysis and gene expression pattern analysis

Differential expression analysis was performed using the R package edgeR (ver. 4.6.1; University of Melbourne). In total, 10 pairwise comparisons were conducted among the five developmental stages (MG, LG, EL, PL, and W2). All comparisons were defined according to the written order, with the earlier stage representing expression relative to the later stage. Specifically, the following comparisons were analyzed: LG vs. MG, EL vs. MG, PL vs. MG, W2 vs. MG, EL vs. LG, PL vs. LG, W2 vs. LG, PL vs. EL, W2 vs. EL, and W2 vs. PL. Transcripts were considered differentially expressed when the adjusted p-value (*p*.adj) was less than 0.05 and the absolute value of log2 fold change was ≥2. Heatmaps showing the expression patterns of all differentially expressed transcripts (DETs) were generated using the R package pheatmap (ver. 1.0.12). Line plots illustrating gene expression trends across stages were created with the R package TCseq (ver. 1.32.0). The number of DETs among different comparison groups was calculated using the R package UpSetR (ver. 1.4.0; Harvard Medical School).

### Weighted gene co-expression network analysis

To identify genes associated with specific developmental stages, weighted gene co-expression network analysis (WGCNA) was performed using the WGCNA package (ver. 1.73; University of California). After filtering missing values with the goodSamplesGenes function, Pearson correlation coefficients were computed, and a soft-thresholding power was selected using pickSoftThreshold. The co-expression network was constructed using the blockwiseModules function (minModuleSize = 100, mergeCutHeight = 0.25). Module eigengenes were calculated using moduleEigengenes and correlated with developmental stages using the Spearman method implemented in cor, with corPvalueStudent employed to calculate the asymptotic p-values for the corresponding correlations. Module–trait relationships were visualized as heatmaps (labeledHeatmap). Gene significance (GS) and module membership (MM, or kME) were first computed using the *signedKME* function, and their relationship was visualized using *verboseScatterplot*. Genes within each target module were then filtered based on the absolute values of GS (|GS|≥0.4, p<0.05) and MM (|MM|≥0.4, p<0.05) to ensure a strong correlation—either positive or negative—with both the module eigengene and the corresponding trait. The filtered genes were subsequently subjected to Gene Ontology (GO) and Kyoto Encyclopedia of Genes and Genomes (KEGG) enrichment analyses to identify the predominant biological processes and pathways represented in each module. Finally, the top 10 genes with the highest absolute GS and MM values in each module were defined as hub genes.

### Gene Ontology and Kyoto Encyclopedia of Genes and Genomes pathway enrichment analysis and mammary function–related regulatory network construction

Functional enrichment analysis was performed using the clusterProfiler package (ver. 4.16.0) for both differentially expressed genes (DEGs) identified across developmental stages and genes from WGCNA-derived modules significantly associated with stage transitions, within a time-series framework (MG → LG → EL → PL → W2). This approach enabled the characterization of dynamic expression trajectories (captured by DEGs and fuzzy C-means clustering) as well as stage-related co-expression patterns (captured by WGCNA modules). GO terms and KEGG pathways with FDR<0.05 were considered significantly enriched. The background gene set included all annotated *Sus scrofa* genes in the GO and KEGG databases. Genes related to mammary development, apoptosis, immunity, and metabolism were further analyzed for protein–protein interactions (PPIs) using the STRING database (ver. 12.0). Interactions from all available evidence sources with combined score ≥0.4 (medium confidence) were retained, and those below this threshold were excluded. The resulting PPI network was imported into Cytoscape (ver. 3.10.3) for visualization. Network topology parameters were calculated using the NetworkAnalyzer plugin in Cytoscape, and degree centrality (node connectivity) was used as the primary metric. Genes with degree values ranking in the top 10%–50% of the network were considered hub genes to capture both highly connected core regulators and moderately connected but potentially important genes.

### Real-time quantitative reverse transcription polymerase chain reaction analysis

Gene-specific primers were designed using Primer-BLAST (NCBI) with melting temperatures set between 55°C–65°C, GC contents of 40%–60%, and amplicon lengths of 100–300 bp to ensure specificity and amplification efficiency. Total RNA was extracted with TRIzol reagent (Invitrogen), and reverse transcription coupled with polymerase chain reaction (PCR) amplification was performed using the One Step TB Green PrimeScript RT-PCR Kit (Takara). Real-time quantitative reverse transcription polymerase chain reaction (RT-qPCR) reactions were conducted on a LightCycler 96 system (Roche). Primer efficiency was verified by standard curves generated from serially diluted cDNA, and only primers with efficiencies between 90% and 105% were used. For data normalization, reference genes were selected using NormFinder from 15 candidates, and the geometric mean of GAPDH and TPT1 was applied as the internal control. Relative expression levels were calculated using the modified Pfaffl method (specific calculation steps at https://toptipbio.com/qpcr-multiple-reference-genes/) [10]. Primer sequences and efficiencies are listed in Supplement 3.

### Statistical analysis

One-way ANOVA tested gene expression differences across developmental stages. p<0.05 was considered significant. Tukey’s HSD was used for post-hoc comparisons. All analyses and graphs were performed using R (ver. 4.5.0).

## RESULTS

### Basic statistical analysis of sequencing results

**Figure 1:**
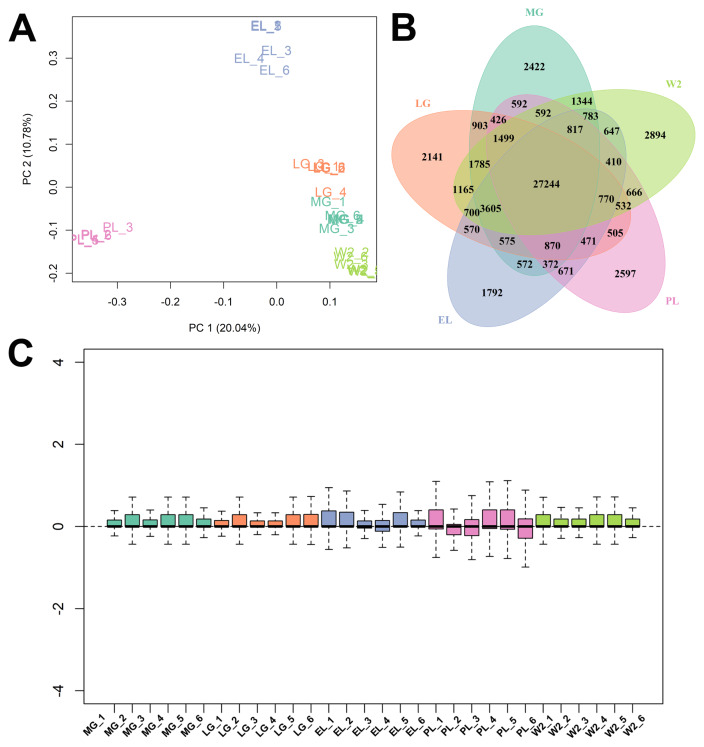
Quality control and gene expression overview across mammary gland stages. (A) Principal component analysis (PCA) plot showing sample clustering at five stages: mid-gestation (MG), late gestation (LG), early lactation (EL), peak lactation (PL), and early involution (W2). PC1 and PC2 explain 20.04% and 10.78% of the variance. (B) Venn diagram showing 27,244 transcripts commonly expressed across all stages. (C) Box plots of normalized gene expression levels for each sample.

Transcriptome sequencing yielded an average of 10.51 million clean reads per library, with alignment rates exceeding 92% (Supplement 4). PCA revealed dispersed sample distribution prior to normalization, while normalization markedly improved within-group clustering and intergroup separation (Figure 1A and Supplement 5). In total, 61,773 non-redundant expressed transcripts (FPKM≥0.5) were expressed, of which 27,244 were commonly detected across all groups (Figure 1B and Supplement 6). Boxplot analyses demonstrated that normalization enhanced the consistency of gene expression distributions and reduced sample variability, thereby improving data reliability and comparability (Figure 1C).

### Expression characteristics of highly expressed genes in mammary glands across five physiological stages

**Figure 2:**
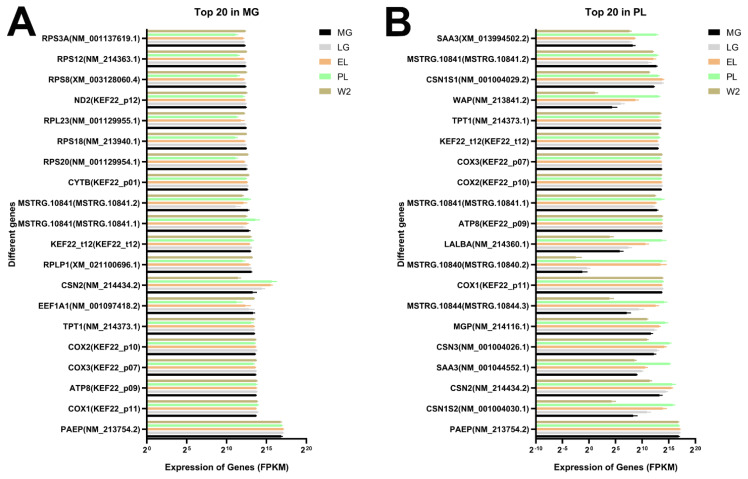
Expression profiles of highly abundant genes in mammary tissues at different physiological stages. (A) Expression distribution (FPKM values) of the top 20 most highly expressed genes identified at the mid-gestation (MG) stage across the five stages: MG, late gestation (LG), early lactation (EL), peak lactation (PL), and early involution (W2; Day 2 after weaning). (B) Expression distribution (FPKM values) of the top 20 most highly expressed genes identified at the PL stage across the five stages: MG, LG, EL, PL, and W2.

To investigate transcript abundance across stages, we identified the top 20 highly expressed transcripts in each group (MG, LG, EL, PL, and W2) (Figures 2A, 2B, Supplement 7).

At the MG stage, ribosomal (e.g., *RPS3A*, *RPS12*) and mitochondrial transcripts (e.g., *ND2*, *COX1–3*, *ATP8*) were predominant, indicating active protein synthesis and energy metabolism. Early high expression of *CSN2* and *PAEP* suggested preparatory activation for lactation.

During LG, transcripts related to milk protein synthesis (e.g., *CSN1S1*, *CSN2*), translation (*RPLP1*, *EEF1A1*, *TPT1*), and energy metabolism (*COX* genes) were enriched, reflecting epithelial cell proliferation and secretory readiness.

In EL, sharp upregulation of casein genes (*CSN1S1*, *CSN1S2*, *CSN2*, *CSN3*) and *FABP3* indicated robust activation of milk production and lipid metabolism. High expression of ribosomal and translation factors further supported intense protein synthesis.

At the PL stage, expression of milk protein genes (e.g., *CSN1S1–3*, *LALBA*, *WAP*), mitochondrial transcripts, and immune-related genes (*SAA3* isoforms, *PAEP*) peaked, reflecting maximal secretory and metabolic activity. Novel transcripts (e.g., *MSTRG.10840*, *MSTRG.10841*) were also highly expressed, suggesting uncharacterized roles in lactation.

In W2, transcripts associated with mitochondrial function (e.g., *COX1–3*, *ATP6*, *ND2*), protein synthesis (ribosomal genes, *EEF1A1*), and stress adaptation (*TPT1*, *PAEP*) remained abundant, indicating sustained biosynthesis and cellular remodeling during W2.

Overall, dynamic shifts in transcript expression across stages reflected distinct biological priorities, including preparation for lactation, secretory activation, and tissue remodeling.

### Differential gene expression analysis and validation by real-time quantitative reverse transcription polymerase chain reaction

**Figure 3:**
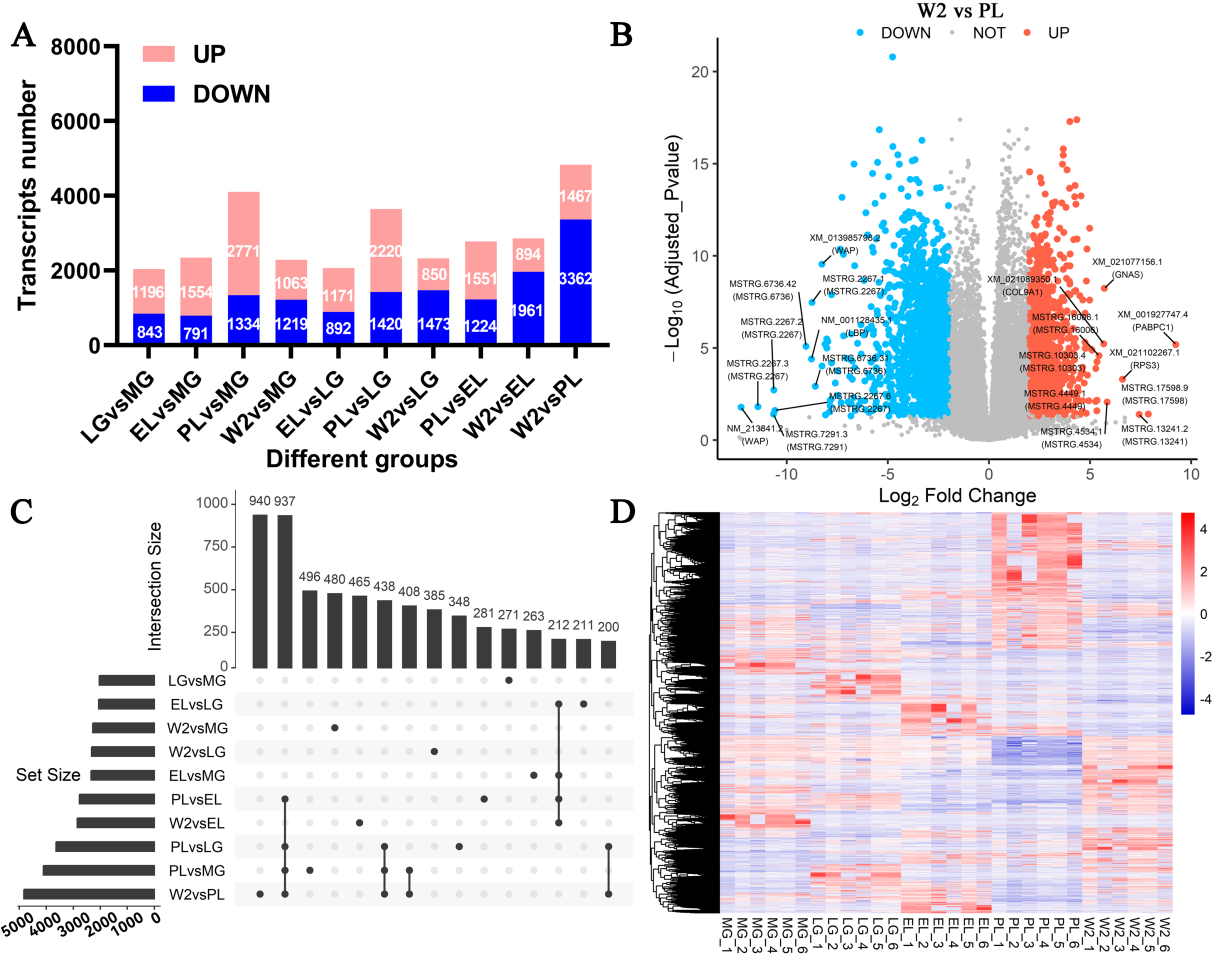
Analysis of differentially expressed transcripts (DETs) across mammary gland stages. (A) Numbers of upregulated and downregulated transcripts in each pairwise comparison among five stages: MG, LG, EL, PL, and W2. (B) Volcano plot of DETs between W2 and PL; red: upregulated, blue: downregulated, grey: not significant. (C) Intersection analysis of DETs across all pairwise comparisons using an UpSet plot. The horizontal bars (left) represent the total number of DETs detected in each comparison (set size). The vertical bars (top) indicate the number of DETs shared among different comparisons (intersection size). The connected black dots (bottom matrix) illustrate the specific overlap patterns among comparisons. This plot highlights both unique and shared DETs across different developmental transitions. (D) Heatmap of all DETs showing distinct expression clusters across stages (red: high, blue: low expression). LG, late gestation; MG, mid-gestation; EL, early lactation; PL, peak lactation; W2, early involution.

Differential expression analysis identified 12,239 transcripts with significant changes across five lactation stages (Figure 3A, Supplement 8). The comparison between W2 and PL showed the greatest number of DETs, totaling 4,829, including 1,467 upregulated and 3,362 downregulated transcripts. Fewer DETs were observed in EL vs LG and LG vs MG comparisons, indicating more pronounced transcriptional remodeling during the transition from lactation to involution.

The volcano plot of the W2 vs PL group (Figure 3B) highlighted significant upregulation of transcripts such as *GNAS*, *COL9A1*, *PABPC4*, and *RPS3*, while key lactation-related genes, including *WAP*, *LBP*, and novel transcripts (e.g., *MSTRG.2267*), were downregulated, consistent with mammary gland involution and cessation of milk secretion.

Upset analysis revealed 937 transcripts shared across multiple comparisons, with additional smaller overlaps among other group pairs (Figure 3C). Heatmap clustering of all DETs demonstrated distinct stage-specific expression patterns (Figure 3D), reflecting dynamic transcriptional regulation during mammary development and involution.

To validate RNA-seq findings, RT-qPCR was performed on ten representative DETs (*LSAMP*, *BHLHE40*, *SPP1*, *UNG*, *DNAJC8*, *NCBP2*, *STAT5B*, *LZTS1*, *TCN1*, and *VDR*) across all stages (Supplement 9). Expression trends from RT-qPCR showed high concordance with RNA-seq data, confirmed by a strong Pearson correlation (R = 0.87, p<2.2e-16) (Supplement 9C). These results support the robustness and reliability of the transcriptomic analysis.

### Expression pattern analysis of differentially expressed genes

**Figure 4:**
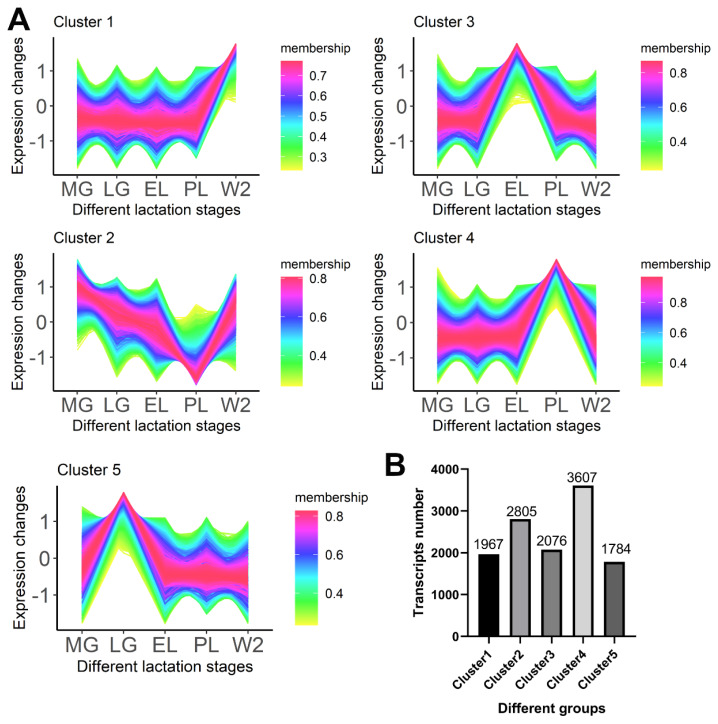
Clustering of expression patterns for differentially expressed transcripts (DETs). (A) Fuzzy C-means clustering grouped DETs into five clusters (Cluster 1–5) based on normalized expression trajectories across MG, LG, EL, PL, and W2. Each line represents one transcript; color intensity reflects membership strength (magenta = high). (B) Transcript counts per cluster. Cluster 4 had the most transcripts (3,607), and Cluster 5 had the fewest (1,784). MG, mid-gestation; LG, late gestation; EL, early lactation; PL, peak lactation; W2, early involution.

Fuzzy C-means clustering identified five distinct transcript clusters with unique expression profiles across lactation stages (Figure 4, Supplement 10), reflecting dynamic gene expression changes in the mammary gland throughout the lactation cycle. Most transcripts showed high membership probabilities (>0.7), supporting the reliability of the clustering results (Figure 4A).

Cluster 1 transcripts peaked in expression at W2 and were significantly enriched in functions related to cytoskeleton organization, signal transduction, and metabolic regulation (Supplements 11A, 11B, Supplement 12), suggesting roles in mammary tissue remodeling during the dry period.

Cluster 2 transcripts were highly expressed during mid-pregnancy, then declined towards PL. Functional enrichment indicated involvement in cell cycle regulation, proliferation, and structural remodeling (Supplements 13A, 13B), reflecting the physiological transition from cell growth to differentiation.

Cluster 3 showed elevated expression during EL, with genes associated with autophagy, mitochondrial regulation, and cell cycle processes (Supplements 14A, 14B), implying active cellular renewal and metabolic adjustment during this stage.

**Figure 5:**
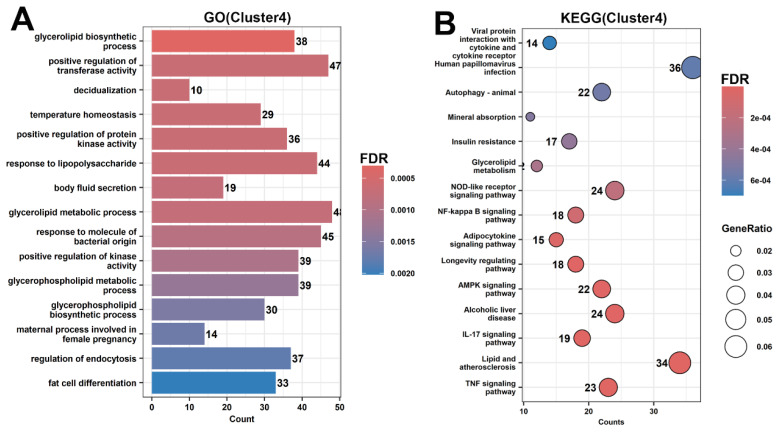
Gene Ontology (GO) and Kyoto Encyclopedia of Genes and Genomes (KEGG) functional enrichment analysis of differentially expressed transcripts in Cluster 4. (A) GO analysis: the X-axis shows the number of enriched transcripts; color indicates significance (FDR), with warmer colors representing higher significance. (B) KEGG pathway analysis: circle size reflects the gene ratio (GeneRatio), and color denotes enrichment significance (FDR).

Cluster 4 transcripts peaked at PL and were enriched in pathways related to lipid metabolism, energy production, and immune response (Figures 5A, 5B), consistent with the high metabolic demands of milk synthesis.

Cluster 5 transcripts exhibited highest expression during late pregnancy, enriched in autophagy, chromatin modification, and cytoskeletal remodeling pathways (Supplements 13B, 15A), likely preparing the mammary gland for lactation onset.

Cluster 4 contained the largest number of transcripts (3,607), followed by Cluster 2 (2,805) and Cluster 5 (1,784) (Figure 4B). These findings reveal complex, stage-specific transcriptomic regulation during mammary gland development and lactation.

### Identification of co-expression modules associated with different stages of mammary gland development

**Figure 6:**
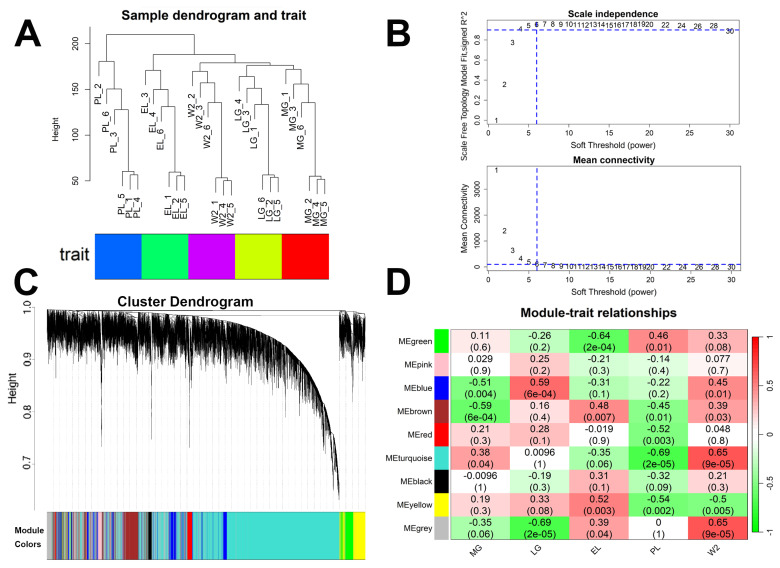
Weighted gene co-expression network analysis (WGCNA) of mammary tissues across different lactation stages. (A) Sample clustering tree based on hierarchical clustering; colors indicate stages: MG, LG, EL, PL, and W2. (B) Soft-thresholding power analysis. Left: scale-free topology fit index (R^2^) vs. power; right: mean connectivity vs. power. (C) Gene dendrogram showing module clustering; modules are color-coded. (D) Heatmap of module–trait correlations. Cells show correlation coefficients between modules and stages, with p-values in parentheses; red = positive, blue = negative correlation. MG, mid-gestation; LG, late gestation; EL, early lactation; PL, peak lactation; W2, early involution.

WGCNA was conducted to explore coordinated gene expression patterns across five stages of mammary gland development (MG, LG, EL, PL, and W2) (Supplements 16–26). Sample clustering confirmed clear stage-specific grouping with no outliers (Figure 6A). A soft-thresholding power of six was selected to achieve a scale-free topology (Figure 6B). Based on this, 15,000 transcripts were clustered into nine distinct modules (Figure 6C), with the gray module representing unassigned genes.

Module–trait correlation analysis identified stage-specific associations (Figure 6D). At MG, significant negative correlations were observed for the blue (r = −0.51, p = 0.004) and brown modules (r = −0.59, p = 6×10^−4^). At LG, the blue module showed a significant positive correlation (r = 0.59, p = 6×10^−4^). At EL, significant associations included a negative correlation for the green module (r = −0.64, p = 2×10^−4^) and a positive correlation for the yellow module (r = 0.52, p = 0.003). At PL, significant negative correlations were detected for the red (r = −0.52, p = 0.003), turquoise (r = −0.69, p = 2×10^−5^), and yellow modules (r = −0.54, p = 0.002). At W2, the turquoise module was positively correlated (r = 0.65, p = 9×10^−5^), whereas the yellow module was negatively correlated (r = −0.50, p = 0.005). Modules with |r|≥0.5 and p<0.05 were considered significantly associated with specific developmental stages.

These findings suggest that distinct co-expression modules are associated with specific physiological stages and may participate in key regulatory processes during mammary gland development and lactation.

### Functional analysis of genes within co-expression modules

**Figure 7:**
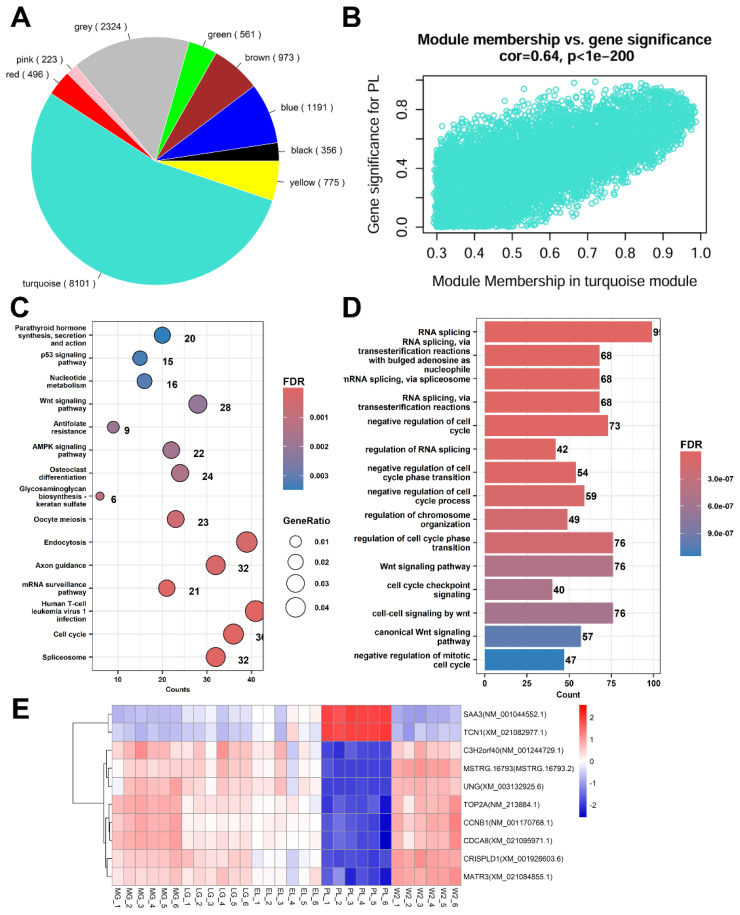
Analysis of the turquoise module associated with peak lactation (PL). (A) Pie chart showing gene counts in each of the nine modules; the turquoise module contained the most genes (8,101). (B) Positive correlation between module membership (MM) and gene significance (GS) for PL in the turquoise module (r = 0.64, p<1e-200). (C) Kyoto Encyclopedia of Genes and Genomes (KEGG) pathway enrichment of turquoise module genes; key pathways include spliceosome, cell cycle, Wnt signaling, endocytosis, and AMPK signaling. Circle size = gene ratio; color = FDR. (D) Gene Ontology (GO) biological process enrichment; genes mainly involved in RNA splicing, cell cycle checkpoint, and chromosome organization. (E) Heatmap of top 10 hub genes in the turquoise module across MG, LG, EL, PL, and W2 (red = high, blue = low expression). MG, mid-gestation; LG, late gestation; EL, early lactation; PL, peak lactation; W2, early involution.

To interpret the biological significance of the co-expression modules, functional enrichment analysis was performed for genes within each module (Supplements 16–26). The turquoise module (8,101 genes) showed a strong negative correlation with the PL stage (r = −0.69, p = 2×10^−5^) (Figures 6D, 7A). GO and KEGG enrichment analyses indicated enrichment in RNA splicing, cell cycle regulation, AMPK, and Wnt signaling pathways (Figures 7C, 7D). Hub genes identified based on module eigengene and GS-MM correlation included *SAA3*, *TCN1*, *TOP2A*, *CCNB1*, *CDCA8*, and others (Figures 7B, 7E). Notably, *SAA3* and *TCN1* were significantly upregulated during PL and may contribute to immune modulation and epithelial metabolism, respectively. Downregulation of *CCNB1*, *CDCA8*, *MATR3*, and *UNG* suggests a shift from cell proliferation to secretory activity during PL.

The brown module, associated with MG (r = −0.59, p = 6×10^−4^), contained genes involved in cell proliferation and tissue remodeling, including myeloid cell differentiation and mesenchymal proliferation (Supplements 21A, 21B). KEGG analysis showed enrichment in the cell cycle, ErbB, and axon guidance pathways (Supplement 21C). Hub genes such as *STAT5B*, *VDR*, and *RC3H1* exhibited stage-specific expression patterns (Supplement 21D), supporting their roles in mammary morphogenesis during gestation.

The blue module, correlated with LG (r = 0.59, p = 6×10^−4^), was enriched in DNA replication initiation, immune modulation, and apoptotic signaling (Supplements 22A, 22B). KEGG pathways included the cell cycle, autophagy, phospholipase D, and estrogen signaling (Supplement 22C). Key genes such as *DDX60*, *CDK18*, and *UNKL* were upregulated in LG, suggesting involvement in epithelial maturation and maternal-fetal adaptation (Supplement 22D).

The green module, positively associated with EL (r = −0.64, p = 2×10^−4^), showed enrichment in secretion regulation, response to nutrients and vitamin D, and hormone signaling (Supplements 23A–23C). KEGG pathways included estrogen, Toll-like receptor, lipid metabolism, and GH signaling. Hub genes like *S100A6*, *ANXA8*, and *CXCL16* were significantly upregulated, indicating their contribution to immune activation, milk secretion, and tissue remodeling (Supplement 23D).

In W2, the turquoise module showed a moderate positive correlation (r = 0.65, p = 9×10^−5^) (Supplement 24A). Genes were enriched in RNA splicing, Wnt signaling, and chromatin remodeling (Supplements 24B, 24C), reflecting transcriptional reprogramming and immune activation during regression. Hub genes such as *PGLYRP1*, *MAP2K3*, and *NCALD* were upregulated, potentially regulating inflammation and stress responses (Supplement 24D). Additional modules associated with W2 included the yellow (Supplement 25) and blue (Supplement 26) modules.

### Analysis of key genes involved in mammary gland development

**Figure 8:**
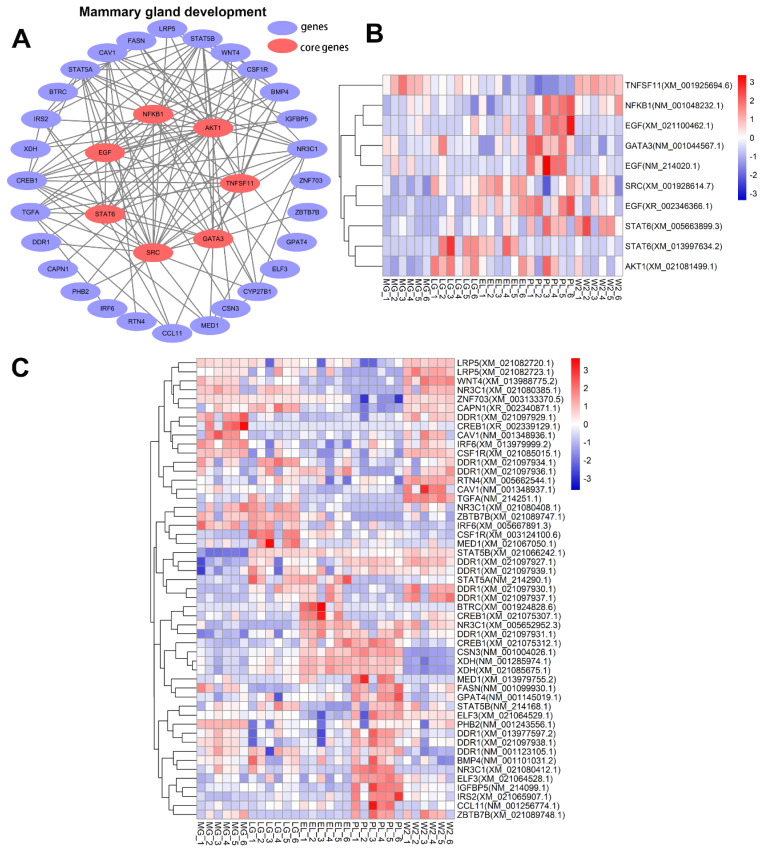
Regulatory network and expression patterns of key genes associated with mammary gland development. (A) Core network showing key genes (*EGF*, *AKT1*, *SRC*, *STAT6*, *GATA3*, *NFKB1*, *TNFSF11*) in red; blue nodes indicate other genes; edges denote regulatory links. (B) Heatmap of core genes with standardized expression (blue: low, red: high). (C) Heatmap of other genes across developmental stages. MG, mid-gestation; LG, late gestation; EL, early lactation; PL, peak lactation; W2, early involution.

Integrating differential expression and WGCNA results with pathway enrichment analysis, we identified seven core genes—*EGF*, *AKT1*, *SRC*, *GATA3*, *STAT6*, *TNFSF11*, and *NFKB1*—that form the central nodes of a regulatory network potentially governing mammary gland development (Figure 8A). These genes interact with 28 additional regulators and are likely involved in coordinating mammary epithelial proliferation, differentiation, and remodeling. Heatmap analysis further revealed stage-specific expression patterns (Figure 8B). *EGF* was upregulated during early and PL, promoting epithelial activation; *AKT1* showed stable elevation from LG to PL, supporting survival and metabolic activity. *SRC* peaked at lactation onset, likely mediating cytoskeletal remodeling. *GATA3* was enriched during PL, consistent with its role in epithelial maintenance. *STAT6* increased moderately during lactation, suggesting involvement in immune adaptation. *TNFSF11* showed higher expression during gestation and W2, indicating a role in tissue remodeling. *NFKB1* was elevated at PL and W2, linking inflammatory signaling to functional restructuring of the gland.

### Analysis of key genes associated with mammary gland remodeling

**Figure 9:**
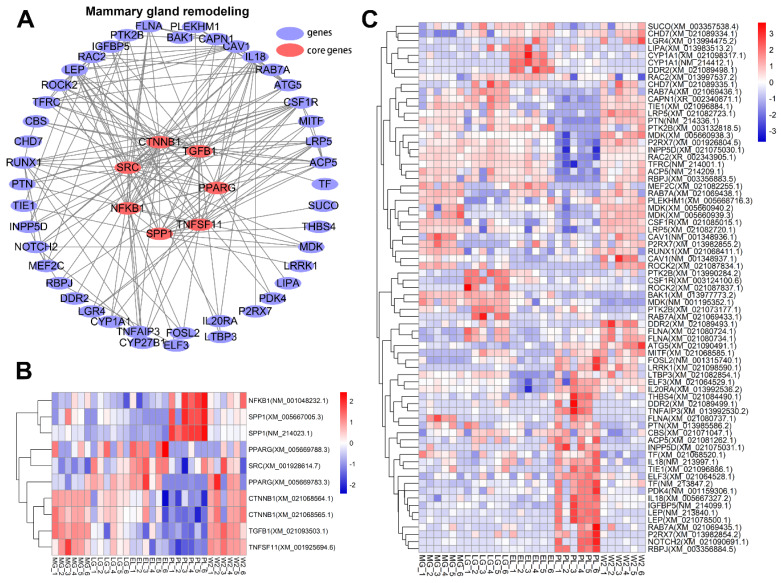
Regulatory network and expression patterns of key genes associated with mammary gland remodeling. (A) Core regulatory network highlighting key genes (red nodes: *NFKB1*, *SPP1*, *PPARG*, *SRC*, *CTNNB1*, *TGFB1*, *TNFSF11*). Blue nodes represent other related genes; edges indicate predicted interactions. (B) Heatmap of core genes showing standardized expression across samples (blue: low, red: high). (C) Heatmap of other network genes across lactation stages. MG, mid-gestation; LG, late gestation; EL, early lactation; PL, peak lactation; W2, early involution.

To further elucidate the molecular basis of mammary gland remodeling, we examined key genes exhibiting marked expression changes during W2 (day 2 post-weaning). The chromatin remodeler *CHD7* was significantly upregulated, suggesting its role in transcriptional reprogramming. Genes involved in vesicle trafficking and endocytosis, such as *RAB7A* and *CAV1*, were also elevated, likely facilitating apoptotic debris clearance. Upregulation of *ATG5* and *CAPN1* indicated activation of autophagy and proteolysis pathways. Immunoregulatory genes including *CSF1R*, *FOSL2*, and *IL20RA* were increased, reflecting enhanced immune response. These changes highlight a coordinated activation of immune, apoptotic, and structural remodeling programs essential for mammary gland regression and regeneration (Figure 9C).

### Analysis of apoptosis-related genes in mammary gland tissue

To characterize the molecular expression features of apoptosis in mammary gland tissue, a regulatory network was constructed comprising 11 core genes (*AKT1*, *CTNNB1*, *RELA*, *GSK3B*, *SRC*, *PTGS2*, *ICAM1*, *CFLAR*, *BCL2*, *BCL2L1*, and *NFE2L2*) and 82 associated genes (Supplement 27A). The anti-apoptotic gene *BCL2* exhibited high expression during the MG, LG, and EL stages, but its expression decreased markedly at the W2 stage (Supplement 27B). In contrast, core genes such as *GSK3B*, *NFE2L2*, *RELA*, and *CTNNB1* showed low expression during the early and PL stages but were highly expressed during the dry period and gestation stages. Additionally, the expression patterns of the 82 apoptosis-related genes are presented in the heatmap shown in Supplement 27C.

### Analysis of mammary gland immune-related genes

To characterize immune-related molecular expression in the mammary gland, a gene regulatory network was constructed (Supplement 28), identifying 21 core genes (e.g., *AKT1*, *MYD88*, *TLR2*, *IKBKB*, *RELA*, *TNFAIP3*, *CD14*, *CD36*, *CAV1*, *PIK3R1*) and 40 associated genes. Heatmap analysis showed dynamic expression patterns across physiological stages. During early and PL, genes such as *CD36, AKT1, IKBKB, BIRC3, XIAP*, and *TNFAIP3* were notably upregulated, indicating active immune engagement during milk production. In contrast, *CAV1, PIK3R1, TRAF3, MAP3K7*, and *TBK1* were highly expressed in W2, suggesting enhanced immune signaling associated with tissue remodeling. Some genes (*TLR2, SRC, CD36, CAV1, LYN*) showed gradual upregulation from late pregnancy through lactation, implicating roles in pathogen recognition, lipid metabolism, and signal transduction. These stage-specific expression profiles of immune genes highlight the shifting immunological priorities of the mammary gland, balancing defense, milk secretion, and involution. The findings offer new insights into immune regulation during functional transitions of the sow mammary gland.

### Analysis of lactation-related genes in the mammary gland

To investigate genes involved in lactation regulation, a gene regulatory network associated with mammary lactation was constructed (Supplement 29). The network comprised seven core genes (*STAT5A*, *CSN3*, *CREB1*, *CYP27B1*, *OAS2*, *MED1*, and *STAT5B*) and eight associated genes. Heatmap analysis showed that *STAT5A* and *STAT5B* were significantly upregulated during early (EL) and PL, indicating key roles in epithelial differentiation and lactation initiation. *CSN3* was markedly increased in EL and PL, reflecting its role in milk protein synthesis and secretory activity. *CREB1* and *MED1* showed elevated expression from LG to EL, suggesting involvement in transcriptional regulation for lactation onset. *OAS2* peaked at PL, potentially linked to immune or antiviral responses during lactation. These dynamic expression patterns highlight the importance of these genes in regulating lactation and provide a basis for further mechanistic studies.

### Analysis of genes related to substance synthesis and metabolism in the mammary gland

To investigate substance synthesis and metabolic regulation in the mammary gland, a gene regulatory network was constructed (Supplement 30). The network comprised ten core genes (*AKT1*, *LEP*, *STAT3*, *GSK3B*, *SRC*, *IGF1*, *TGFB1*, *NFKB1*, *EGF*, and *IGF2*) and 23 associated genes. Heatmap analysis showed distinct expression patterns of core genes across physiological stages. Notably, *IGF2, AKT1, EGF, NFKB1, STAT3*, and *LEP* were significantly upregulated at PL, indicating key roles in supporting active synthesis and metabolism. Conversely, *GSK3B, SRC, TGFB1*, and *IGF1* exhibited variable expression during LG and W2, suggesting involvement in epithelial cell proliferation, apoptosis, and remodeling. These results emphasize coordinated regulation by core genes in mammary gland function and provide a molecular basis for further study of lactation mechanisms.

## DISCUSSION

The mammary gland’s development, lactation, and involution are tightly regulated by complex molecular networks. Our study profiled transcriptomic changes across five key stages in the porcine mammary gland, providing an integrated view of the molecular mechanisms driving these functional transitions. Previous studies largely focused on lactation or involution phases, such as single-cell RNA-seq analyses of porcine mammary tissues at LG and postpartum stages [4], transcriptional comparisons between colostrum and mature milk from sows at different parities [5], and tissue biopsies around parturition [6]. In contrast, this work spans from MG to W2, systematically depicting continuous transcriptional dynamics and employing whole-tissue transcriptomics to capture integrated physiological states (Figures 1, 2).

From MG to LG, porcine mammary tissues exhibited elevated expression of ribosomal protein genes (e.g., *RPS8*, *RPS12*) and mitochondrial metabolism genes (*COX1*, *COX2*), reflecting enhanced protein synthesis and energy metabolism needed for ductal growth and alveolar morphogenesis (Figure 2A). This metabolic activation aligns with prior findings in pigs [4]. Additionally, *PAEP* maintained high expression during gestation, indicating early preparation for secretory functions, consistent with its association with milk traits in cattle [11,12].

During EL and PL stages, milk protein genes (*CSN1S1*, *CSN2*, *CSN3*, *LALBA*, *WAP*) were markedly upregulated, indicating robust secretory activation peaking at PL (Figure 2B). This pattern is consistent with transcriptomic results from goats [13], sheep [14], cattle [15], and pigs [6]. At W2, mitochondrial metabolism and stress-related genes remained highly expressed, suggesting ongoing metabolic activity and adaptive remodeling during involution (Supplement 7).

Clustering analysis categorized DEGs into five distinct patterns corresponding to physiological stages. Cluster 1 genes, highly expressed at W2, were enriched in cytoskeletal remodeling, signal transduction, and metabolic regulation pathways (Figure 4A, Supplement 11), highlighting structural and functional adaptations during W2. This corresponds well with known processes of apoptosis and alveolar remodeling in involution [2], as well as immune activation dynamics reported in epithelial cells during early and late involution phases [4]. Similar immune-related enrichments have been observed in dry-period mammary glands of dairy goats [16]. KEGG pathways identified in Cluster 1 include autophagy and endocytosis, critical for pathogen clearance and tissue homeostasis during involution [17,18].

Cluster 2 genes, upregulated at MG, were enriched in Wnt signaling and cell cycle pathways, indicative of rapid proliferation and tissue expansion (Supplement 7A), consistent with Wnt’s established role in early mammary morphogenesis [19]. Enrichment of chromosome segregation and mitotic processes suggests synchronized cellular growth and architectural remodeling required for subsequent lactation [20].

Cluster 3 genes, upregulated at EL, involve Wnt signaling, autophagy regulation, and G1/S cell cycle transition (Figures 4, 5). Autophagy supports cellular homeostasis and organelle renewal during the shift to a secretory phenotype, promoting Golgi and ER function critical for milk biosynthesis and secretion [21]. It also supplies metabolic substrates to meet increased demands during lactation initiation [22].

Our results demonstrate that genes related to lipid metabolism and energy regulation are upregulated at PL (Figures 4, 5), consistent with previous studies reporting increased metabolic demands during high milk production [23]. The enrichment of genes involved in cytoskeletal remodeling and chromatin modification during LG aligns with earlier findings that morphological and epigenetic changes prepare the mammary gland for lactation (Supplement 15). Additionally, activation of ubiquitin-mediated proteolysis, autophagy, and lysosomal pathways supports the observations on the importance of organelle turnover and protein homeostasis in mammary tissue remodeling [24]. Collectively, these findings further confirm the coordinated regulation of metabolic and structural processes during mammary gland functional transitions, providing a molecular basis for adaptation throughout the lactation cycle.

Network analysis identified seven core regulatory genes: *EGF*, *AKT1*, *SRC*, *GATA3*, *STAT6*, *TNFSF11*, and *NFKB1*. *EGF* showed sustained upregulation during EL and PL, playing a key role in epithelial proliferation and lactation initiation alongside endocrine hormones [25,26]. It is known that during lactation initiation, progesterone and estrogen levels decline, while prolactin, insulin, and cortisol increase and act synergistically with EGF to shift mammary epithelial cells from a proliferative to a secretory state [27]. At PL, prolactin, IGF-1, insulin, and cortisol remain elevated, with EGF continuing to cooperate with these hormones to ensure epithelial survival, secretory activity, and milk production [25]. This indicates that EGF serves as a core regulatory factor that, through stage-specific cooperation with key endocrine hormones, drives lactation initiation and sustains secretory stability at PL, thereby acting as a pivotal mediator of mammary development and functional transition.

*AKT1* expression rose in LG and PL, supporting cell survival, metabolism, and anti-inflammatory responses [28,29]. *SRC* promoted cytoskeletal remodeling and functional activation at lactation onset [30]. *GATA3* maintained epithelial differentiation and secretory phenotype stability [31]. *STAT6*, mediating IL-4/IL-13 signaling, likely regulates immune environment and tissue repair during lactation and W2 [32]. Together, these hub genes coordinate epithelial proliferation, differentiation, and remodeling, forming a molecular network that drives mammary gland development and function.

Nevertheless, some limitations should be acknowledged. Transcriptomic profiling provides valuable insights into gene expression dynamics but cannot establish direct causal relationships. Functional validation, such as gene knockdown or overexpression in mammary epithelial cells, will be necessary to confirm the regulatory roles of the identified hub genes. In addition, bulk-tissue RNA-seq captures integrated physiological states but obscures cell type–specific transcriptional heterogeneity, which could be further resolved by single-cell or spatial transcriptomics in future studies.

## CONCLUSION

To our knowledge, this study represents the most comprehensive transcriptomic profiling of porcine mammary glands across five key physiological stages: MG, LG, EL, PL, and W2. Distinct molecular features were identified at each stage, including active cell proliferation and mitochondrial metabolism during gestation; upregulation of milk protein genes (*CSN1S1*, *CSN2*, *LALBA*) and lipid metabolism at lactation initiation; enhanced metabolic and signaling pathways such as glycerolipid metabolism and NF-κB at PL; and increased autophagy, apoptosis, and immune response during W2. Key regulators such as *STAT5A/B*, *AKT1*, and *CTNNB1* were highlighted by gene co-expression and protein interaction analyses. These results reveal the dynamic gene regulatory network underlying mammary gland development and function, offering targets to improve sow lactation and genetic selection. To build on these findings, future studies will validate the roles of key candidate genes and pathways using cultured porcine mammary epithelial cells and mammary organ models.

## Data Availability

The data supporting this study are available from the corresponding author upon reasonable request. RNA-seq data are accessible via NCBI GEO (accession: GSE295679).

## SUPPLEMENTARY MATERIAL

**Supplement 1**. Animal ethics approval statement.

**Supplement 2**. Summary of sample collection time points, animal characteristics, and tissue sampling sites.

**Supplement 3**. Primer sequences for quantitative PCR and stability evaluation of candidate housekeeping genes using NormFinder analysis.

**Supplement 4**. Summary of total reads, clean reads, mapped reads, and mapping rates of mammary gland samples across developmental stages.

**Supplement 5**. Pre-normalization quality assessment of transcriptomic data across different mammary gland developmental stages.

**Supplement 6**. Raw counts of all transcripts across different mammary gland developmental stages.

**Supplement 7**. Expression profiles of the top 20 highly expressed genes at different mammary gland developmental stages.

**Supplement 8**. Differentially expressed transcripts identified across different developmental stages of the mammary gland.

**Supplement 9**. Validation of RNA-Seq results by RT-qPCR analysis.

**Supplement 10**. Clustering analysis of differentially expressed transcripts based on their expression profiles.

**Supplement 11**. Functional enrichment analysis of genes in Cluster 1.

**Supplement 12**. GO and KEGG functional enrichment analyses for each cluster of differentially expressed transcripts.

**Supplement 13**. Functional enrichment analysis of genes in Cluster 2.

**Supplement 14**. Functional enrichment analysis of genes in Cluster 3.

**Supplement 15**. Functional enrichment analysis of genes in Cluster 5.

**Supplement 16**. WGCNA results and correlation analysis between gene co-expression modules and the five developmental stages.

**Supplement 17**. WGCNA analysis and functional annotation of the brown module.

**Supplement 18**. Correlation analysis and functional enrichment of the green module associated with the peak lactation (PL) stage.

**Supplement 19**. Correlation analysis and functional enrichment of the red module associated with the peak lactation (PL) stage.

**Supplement 20**. Correlation analysis and functional enrichment of the yellow module associated with the peak lactation (PL) stage.

**Supplement 21**. Correlation analysis and functional enrichment of the brown module associated with the mid-gestation (MG) stage.

**Supplement 22**. Correlation analysis and functional enrichment of the blue module associated with the late gestation (LG) stage.

**Supplement 23**. Correlation analysis and functional enrichment of the green module associated with the early lactation (EL) stage.

**Supplement 24**. Analysis of the turquoise module highly associated with the W2 stage.

**Supplement 25**. Analysis of the yellow module associated with the W2 stage.

**Supplement 26**. Analysis of the blue module associated with the W2 stage.

**Supplement 27**. Construction and expression analysis of the apoptosis-related core regulatory network.

**Supplement 28**. Construction and expression analysis of the mammary gland immunity-related gene network.

**Supplement 29**. Construction and expression analysis of the lactation-related gene network.

**Supplement 30**. Construction and expression analysis of the substance biosynthesis and metabolism-related gene network.
