# Supplementary material for: Time-resolved transcriptomic profiling of mammary gland tissue during ductal morphogenesis, lactation activation, and involution in sows
Source: Anim Biosci. 2025 Nov 14;39(5):250560. doi: 10.5713/ab.250560 (PMC13175048; doi:10.5713/ab.250560)
Supplement: Supplementary file 1 [file ab-250560-Supplement-1.pdf]

Supplementary Materials

塔里木大学涉及人体及动物（生物）科技伦理实验审查同意书

申请编号	B20250506001	批准编号	PB20250506001

本《涉及人体及动物（生物）科技伦理实验方案》经过塔里木大学科技伦理委员会审核，符合涉及人体及动物（生物）科技伦理保护原则，符合国家实验涉及人体及动物（生物）科技伦理的相关规定。相关信息如下：

文章名称	猪乳腺导管发育、泌乳功能激活与腺体退化过程的时序性转录组研究				
通讯作者	玄荣	职称/学位	讲师/博士	邮箱	xuanrongtarim@taru.edu.cn
第一作者	彭亚南	职称/学位	无/硕士	邮箱	3201124401@taru.edu.cn
审核意见	☑符合涉及人体及动物（生物）科技伦理要求，可以进行申 请项目。<br>☐其他				
	备注：				

塔里木大学科技伦理委员会

塔里木大学科研处（代章）

日　期：2025 年 5 月 6 日

Supplement 1. Animal ethics approval statement.
