# Supplementary material for: Time-resolved transcriptomic profiling of mammary gland tissue during ductal morphogenesis, lactation activation, and involution in sows
Source: Anim Biosci. 2025 Nov 14;39(5):250560. doi: 10.5713/ab.250560 (PMC13175048; doi:10.5713/ab.250560)
Supplement: Supplementary file 2 [file ab-250560-Supplement-2.pdf]

Supplement 2. Summary of sample collection time points, animal characteristics, and tissue sampling sites.

Sample Collection Time Point	Age (months)	Body Weight (kg)	Chest Girth (cm)	Body Length (cm)	Backfat Thickness (mm)	Gestation/Lactation Stage Description	Tissue Collection Site
Day 70 of Gestation	24	207.86	110	145	18	Mid-Gestation	1–2 cm Below the Third Pair of Teats on the Left Side
Day 70 of Gestation	25	207.18	112	147	19	Mid-Gestation	1–2 cm Below the Third Pair of Teats on the Left Side
Day 70 of Gestation	25	205.78	114	149	18	Mid-Gestation	1–2 cm Below the Third Pair of Teats on the Left Side
Day 70 of Gestation	24	206.71	116	151	18	Mid-Gestation	1–2 cm Below the Third Pair of Teats on the Left Side
Day 70 of Gestation	25	207.22	110	153	19	Mid-Gestation	1–2 cm Below the Third Pair of Teats on the Left Side
Day 70 of Gestation	24	206.95	112	155	20	Mid-Gestation	1–2 cm Below the Third Pair of Teats on the Left Side
Day 110 of Gestation	26	218.75	114	145	21	Late Gestation	1–2 cm Below the Third Pair of Teats on the Left Side
Day 110 of Gestation	25	217.28	116	147	21	Late Gestation	1–2 cm Below the Third Pair of Teats on the Left Side
Day 110 of Gestation	26	218.62	115	149	20	Late Gestation	1–2 cm Below the Third Pair of Teats on the Left Side
Day 110 of Gestation	26	217.32	116	151	19	Late Gestation	1–2 cm Below the Third Pair of Teats on the Left Side
Day 110 of Gestation	25	217.63	114	153	19	Late Gestation	1–2 cm Below the Third Pair of Teats on the Left Side

Day 110 of Gestation	26	218.97	116	155	21	Late Gestation	1–2 cm Below the Third Pair of Teats on the Left Side
Day 2 Postpartum	25	212.14	114	145	21	Early Lactation	1–2 cm Below the Third Pair of Teats on the Left Side
Day 2 Postpartum	25	212.36	112	147	20	Early Lactation	1–2 cm Below the Third Pair of Teats on the Left Side
Day 2 Postpartum	26	210.67	114	149	20	Early Lactation	1–2 cm Below the Third Pair of Teats on the Left Side
Day 2 Postpartum	26	210.89	116	151	21	Early Lactation	1–2 cm Below the Third Pair of Teats on the Left Side
Day 2 Postpartum	25	212.33	110	153	19	Early Lactation	1–2 cm Below the Third Pair of Teats on the Left Side
Day 2 Postpartum	26	214.27	112	155	20	Early Lactation	1–2 cm Below the Third Pair of Teats on the Left Side
Day 10 Postpartum	26	208.57	114	145	18	Peak Lactation	1–2 cm Below the Third Pair of Teats on the Left Side
Day 10 Postpartum	26	208.91	116	147	19	Peak Lactation	1–2 cm Below the Third Pair of Teats on the Left Side
Day 10 Postpartum	26	210.21	113	149	20	Peak Lactation	1–2 cm Below the Third Pair of Teats on the Left Side
Day 10 Postpartum	25	207.64	112	151	18	Peak Lactation	1–2 cm Below the Third Pair of Teats on the Left Side
Day 10 Postpartum	25	210.17	114	153	19	Peak Lactation	1–2 cm Below the Third Pair of Teats on the Left Side
Day 10 Postpartum	26	209.45	116	155	20	Peak Lactation	1–2 cm Below the Third Pair of Teats on the Left Side

Day 2 Post-weaning	26	210.14	110	145	18	Early Dry Period	1–2 cm Below the Third Pair of Teats on the Left Side
Day 2 Post-weaning	25	210.37	112	147	19	Early Dry Period	1–2 cm Below the Third Pair of Teats on the Left Side
Day 2 Post-weaning	26	210.47	112	149	20	Early Dry Period	1–2 cm Below the Third Pair of Teats on the Left Side
Day 2 Post-weaning	27	210.18	111	151	21	Early Dry Period	1–2 cm Below the Third Pair of Teats on the Left Side
Day 2 Post-weaning	27	208.96	110	153	21	Early Dry Period	1–2 cm Below the Third Pair of Teats on the Left Side
Day 2 Post-weaning	27	211.24	112	155	20	Early Dry Period	1–2 cm Below the Third Pair of Teats on the Left Side
