# Supplementary material for: Time-resolved transcriptomic profiling of mammary gland tissue during ductal morphogenesis, lactation activation, and involution in sows
Source: Anim Biosci. 2025 Nov 14;39(5):250560. doi: 10.5713/ab.250560 (PMC13175048; doi:10.5713/ab.250560)
Supplement: Supplementary file 3 [file ab-250560-Supplement-3.pdf]

Supplement 3. Primer sequences for quantitative PCR and stability evaluation of candidate housekeeping genes using NormFinder analysis.

Supplement 3A. Primer sequences of candidate housekeeping genes.

Gene ID	5' primer	3' primer	Tm（5' primer）	GC%（5' primer）	Tm（3' primer）	GC%（3' primer）
NM_001206359.1(GAPDH)	AGTGAACGGATTTGGCCGC	TCTCATGGTTCACGCCCATC	61.33	57.89	60.11	55
NM_214373.1(TPT1)	TGGACTACCGTGAGGATGGT	CAGTGGCCAGCCAGTTATGA	59.96	55	60.04	55
XM_003356239.4(DNAJC8)	AGAGGGTCAGAAGGCGAGAT	CGCCACCTCACATGAGTCTT	60.03	55	60.04	55
XM_021091108.1(VDR)	CATCCGAGGGAGCAATGTCA	CATAACGGAGGTCTCGTGGG	59.82	55	59.9	60
XM_021072721.1(LZTS1)	GTCCGTCGGTCTTTGTCTGT	GACCCTGACAGTTACACGCA	59.97	55	59.97	55
XM_021066242.1(STAT5B)	GAAAGCCAAGCTTGAACACG	TCTCCGCCAACTTCTCACAC	58.24	50	59.97	55
NM_214023.1(SPP1)	GGGCTGCAGACCAAGGAAA	GTGAGGTCTTCCTCTGTGGC	60.23	57.89	60.04	60
XM_003358764.5(NCBP2)	CCGGAAGCCTCTTCATGGTT	TCCTCCCTGCGCTTAGAGTAT	60.04	55	60.13	52.38
XM_021082977.1(TCN1)	AATACACAACGGCAATCCGC	TGTTAAACACCGCGTCCAGA	59.55	50	59.9	50
NM_001245010.1(BHLHE40)	CCAGTCATCCAGCGGACTTT	TGCACATGAGTGCGTGCAT	60.04	55	60.97	52.63
XM_003132925.6(UNG)	GCAAACAGCCCATCAACTGG	CAGGTGGCAGGGTGATCTTT	60.04	55	59.96	55

NM_001244697.1(LSAMP)	CAACGTCACTGAGGAGCACT	CACCGACCCAGGTCTGAAAA	59.97	55	59.89	55

Supplement 3B. qPCR amplification efficiency and correlation coefficients.

Gene ID	Slope of the standard curve	R2	Efficiency
NM_001206359.1(GAPDH)	-3.412571429	0.972458371	96.35147107
NM_214373.1(TPT1)	-3.351428571	0.981204617	98.78344905
XM_003356239.4(DNAJC8)	-3.495238095	0.989327541	93.24289819
XM_021091108.1(VDR)	-3.287142857	0.993785194	101.4723993
XM_021072721.1(LZTS1)	-3.468571429	0.957421038	94.22410556
XM_021066242.1(STAT5B)	-3.392380952	0.968314792	97.14157165
NM_214023.1(SPP1)	-3.45047619	0.974685103	94.90145013
XM_003358764.5(NCBP2)	-3.517142857	0.995812506	92.45167251
XM_021082977.1(TCN1)	-3.365714286	0.990532187	98.2046103
NM_001245010.1(BHLHE40)	-3.530952381	0.963278429	91.9595442
XM_003132925.6(UNG)	-3.478095238	0.987693154	93.8713753
NM_001244697.1(LSAMP)	-3.408095238	0.985104387	96.5255545

R2 = coefficient of determination of the standard curve (%).

Efficiency is calculated as [(10^(-1/curve slope) – 1) x 100], (%).

Supplement 3C. Expression distribution of 15 housekeeping genes across developmental stages.

Sample	GAPDH	ACTB	ATP5B	S100A6	RPL4	FLOT2	RPL32	HPRT1	B2M	UBC	RPL13A	HMBS	EIF4A2	GUSB	TPT1	Group
MG_1	184.9378	614.6918	515.3144	115.5593	2766.546	10.59076	865.561	44.7272	426.4478	178.5928	2747.8	6.409265	483.0443	27.91332	11137.59	MG
MG_2	200.1188	646.3125	548.6849	102.7888	2601.852	11.771	801.0606	59.99197	541.9239	209.9471	2858.589	8.499912	463.1995	27.74149	11543.1	MG
MG_3	209.5573	514.6583	597.5557	123.7614	2684.369	11.07898	607.3107	72.99445	533.6635	230.7548	2873.74	12.50212	421.8947	27.97998	11794.21	MG
MG_4	199.9786	647.5292	548.209	102.0018	2602.012	11.03765	803.2175	59.66065	541.0766	210.3029	2858.567	9.779641	462.8925	26.71495	11543.48	MG
MG_5	199.4107	648.1891	548.7382	101.8944	2602.084	10.67536	803.2437	60.7144	541.2069	209.6141	2859.365	8.581333	463.9529	27.50858	11543.68	MG
MG_6	204.5751	813.0811	532.7408	67.02617	2354.913	10.51263	933.7343	62.07177	663.0964	220.476	2954.012	8.819787	487.1205	25.69937	11699.65	MG
LG_1	161.4231	528.2965	481.1941	51.23417	2710.601	11.60856	1220.318	35.28144	514.4174	186.636	2799.388	4.289519	349.8556	22.10463	10959.7	LG
LG_2	165.1825	481.6028	470.0981	57.46914	2699.514	9.047106	1392.471	37.64771	552.1988	201.3973	2538.57	5.787394	350.696	19.27862	11425.47	LG
LG_3	164.1228	574.0206	402.3049	52.51321	2533.068	5.627991	1984.407	32.12838	491.3913	178.5566	2611.721	4.09555	301.5556	19.00807	11464.54	LG
LG_4	170.6849	342.2458	523.6693	64.60028	2852.868	9.652164	971.5662	48.37441	651.7325	238.2204	2205.367	8.049324	400.2121	17.35065	11852.78	LG
LG_5	165.5541	481.3179	468.9071	56.06105	2699.263	8.737018	1391.913	37.86498	552.7372	201.1989	2538.553	4.529144	351.0724	20.27232	11425.65	LG
LG_6	166.565	481.1408	470.0131	55.52816	2699.524	8.987122	1392.307	39.0681	552.6587	201.2547	2539.189	5.013666	349.8312	20.16384	11425.94	LG
EL_1	160.864	381.7852	467.1546	25.36622	1719.428	8.850427	696.5848	30.45641	339.4678	197.9363	2089.538	5.071844	271.067	18.76087	10946.95	EL
EL_2	159.9221	382.0813	469.0075	24.16836	1718.813	8.940511	696.4437	29.06386	338.4276	197.7764	2089.332	5.122298	270.2435	19.86662	10946.3	EL
EL_3	175.0789	369.3251	523.946	28.47618	2707.713	14.76278	812.2294	41.30806	349.9395	246.7613	2489.005	6.826115	489.491	22.92618	12125.51	EL
EL_4	146.8793	345.7606	404.731	24.65447	249.607	5.804922	402.9016	21.03029	297.5615	172.8294	1668.896	3.489424	143.3203	15.69904	10316.77	EL
EL_5	160.9572	382.4358	468.5803	24.46501	1718.143	9.484223	696.2118	31.01053	338.8131	197.7488	2088.851	5.69901	271.1559	20.03421	10947.2	EL
EL_6	158.1269	432.4121	475.4284	21.98783	2198.819	7.18147	873.7401	27.91343	368.9429	173.8869	2110.049	4.478028	180.9814	21.12627	10397.82	EL
PL_1	96.28729	449.2242	275.8634	112.0576	1184.502	12.83396	691.7931	15.3315	480.0325	189.7428	980.6696	5.587901	195.6642	11.77155	8636.491	PL
PL_2	70.17715	285.1618	303.5563	29.84765	700.1317	6.661003	308.1673	10.02992	264.7738	118.4043	609.4699	4.69153	148.6962	10.69259	5174.195	PL
PL_3	124.1673	628.3709	300.4246	144.8681	1327.538	13.29045	984.083	18.76652	689.3087	132.7552	1031.679	4.88354	189.6597	14.18157	9521.532	PL
PL_4	96.50829	449.6955	276.679	112.209	1184.736	11.97065	691.1347	15.45688	479.9521	190.5028	980.4007	4.048659	196.1944	12.90442	8636.996	PL
PL_5	96.01842	449.6057	275.9285	111.9539	1184.335	11.95574	691.0704	14.8622	479.5633	190.6121	981.1452	4.728754	195.591	12.10752	8637.251	PL
PL_6	94.91874	435.3469	224.9228	162.6342	1526.668	15.59308	782.5175	16.37459	486.6194	318.9156	1300.677	6.170539	249.7591	10.85655	11215.41	PL

W2_1	169.6896	717.0052	557.4859	109.6733	1772.619	7.454392	1056.432	61.54582	413.6273	212.5603	2986.218	7.084764	351.4268	27.22933	11224.01	W2
W2_2	162.6505	886.6597	530.038	37.87026	370.415	7.884785	1224.791	57.86574	475.9242	192.8097	3219.554	4.703153	515.3267	30.42917	10892.93	W2
W2_3	178.7987	355.8303	576.4178	141.0982	2237.225	11.47407	780.3968	64.94308	383.0439	220.967	2857.596	10.45552	451.5637	23.68656	12455.41	W2
W2_4	169.7397	717.0501	557.8642	109.0497	1772.15	8.24965	1056.346	61.08415	413.0934	212.599	2986.436	6.26313	352.1428	28.56813	11224.51	W2
W2_5	170.5208	717.0957	557.4165	109.1628	1773.023	8.400326	1056.139	60.77502	413.9155	212.5287	2986.29	7.392707	351.9573	28.33535	11224.75	W2
W2_6	167.3075	907.9592	566.8122	149.3121	2710.7	6.565944	1163.359	59.18688	381.4611	223.9165	2881.747	4.283201	88.97591	29.25659	10324.45	W2

Supplement 3D. NormFinder-based stability values and ranking of housekeeping genes.

Stability of a single reference gene calculated by Normfinder			
**Gene_ID**	**Group Difference**	**Group Standard Deviation**	**Stability**
GAPDH	0.63	0.11	0.17
TPT1	0.59	0.09	0.18
ATP5B	0.62	0.21	0.23
UBC	0.58	0.2	0.28
GUSB	0.68	0.17	0.28
HMBS	0.62	0.31	0.3
EIF4A2	0.53	0.48	0.31
ACTB	0.43	0.35	0.32
RPL13A	1.45	0.09	0.33
B2M	0.9	0.17	0.34
RPL32	1.07	0.3	0.37
HPRT1	1.71	0.11	0.41
FLOT2	1.41	0.21	0.43
RPL4	1.01	0.66	0.44
S100A6	2.38	0.45	0.72

Stability of paired reference genes calculated by Normfinder		
**Gene1**	**Gene2**	**Stability**
GAPDH	ATP5B	0.18
GAPDH	UBC	0.17
GAPDH	TPT1	0.16
ATP5B	UBC	0.17
ATP5B	TPT1	0.17

UBC	TPT1	0.23
