# Supplementary material for: Time-resolved transcriptomic profiling of mammary gland tissue during ductal morphogenesis, lactation activation, and involution in sows
Source: Anim Biosci. 2025 Nov 14;39(5):250560. doi: 10.5713/ab.250560 (PMC13175048; doi:10.5713/ab.250560)
Supplement: Supplementary file 4 [file ab-250560-Supplement-4.pdf]

Supplement 4. Summary of total reads, clean reads, mapped reads, and mapping rates of mammary gland samples across developmental stages.

ID	Total_reads	Clean reads	Mapping reads	Mapping rate（%）
MG_1	15359658	13627089	12922568	94.83
MG_2	10209215	9640562	8978255	93.13
MG_3	10955924	9888817	9419098	95.25
MG_4	11228872	10203676	9720022	95.26
MG_5	10749101	9485007	8941516	94.27
MG_6	9380179	8530335	8246275	96.67
LG_1	19735456	17931635	16880841	94.14
LG_2	11181784	10560077	10127114	95.9
LG_3	11951622	10752874	9952860	92.56
LG_4	10008072	8822115	8474524	96.06
LG_5	9926854	9264733	8530966	92.08
LG_6	11035192	9752903	8974621	92.02
EL_1	10437247	9840236	9515508	96.7
EL_2	10374501	9764480	9362183	95.88
EL_3	13126162	11661282	10755200	92.23
EL_4	14561353	13134340	12253026	93.29
EL_5	11475742	10190459	9497508	93.2
EL_6	14064962	12799115	12096444	94.51
PL_1	10612547	9618151	9172831	95.37
PL_2	12095287	11088959	10663143	96.16
PL_3	10056002	9342026	8817004	94.38
PL_4	12073055	11302794	10488993	92.8
PL_5	10110591	9164240	8615302	94.01
PL_6	9273962	8317817	7993422	96.1

W2_1	9949970	9198747	8889669	96.64
W2_2	10927580	9969231	9524603	95.54
W2_3	10349213	9244952	8947265	96.78
W2_4	10059467	9459923	8895166	94.03
W2_5	15883509	14006078	13216135	94.36
W2_6	9675245	8855752	8236735	93.01
