# Supplementary material for: Time-resolved transcriptomic profiling of mammary gland tissue during ductal morphogenesis, lactation activation, and involution in sows
Source: Anim Biosci. 2025 Nov 14;39(5):250560. doi: 10.5713/ab.250560 (PMC13175048; doi:10.5713/ab.250560)
Supplement: Supplementary file 5 [file ab-250560-Supplement-5.pdf]

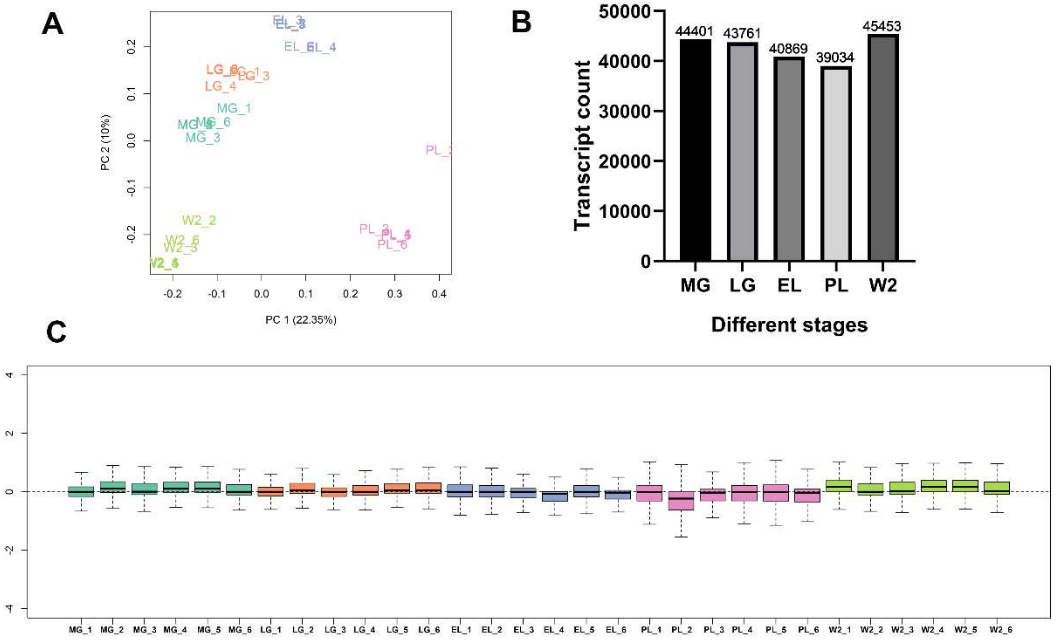

Supplement 5. Pre-normalization quality assessment of transcriptomic data across different mammary gland developmental stages. (A) Principal component analysis (PCA) of raw transcriptomic profiles from five physiological stages: mid-gestation (MG), late gestation (LG), early lactation (EL), peak lactation (PL), and early involution (W2). The first principal component (PC1) and second principal component (PC2) explained 22.35% and 10% of the variance, respectively. (B) Total number of transcripts detected at each stage. W2 samples exhibited the highest transcript count, whereas PL samples showed the lowest. (C) Boxplots of raw expression value distributions across all samples, indicating a generally consistent distribution but with slight sample dispersion before normalization.
