# Supplementary material for: Time-resolved transcriptomic profiling of mammary gland tissue during ductal morphogenesis, lactation activation, and involution in sows
Source: Anim Biosci. 2025 Nov 14;39(5):250560. doi: 10.5713/ab.250560 (PMC13175048; doi:10.5713/ab.250560)
Supplement: Supplementary file 7 [file ab-250560-Supplement-7.pdf]

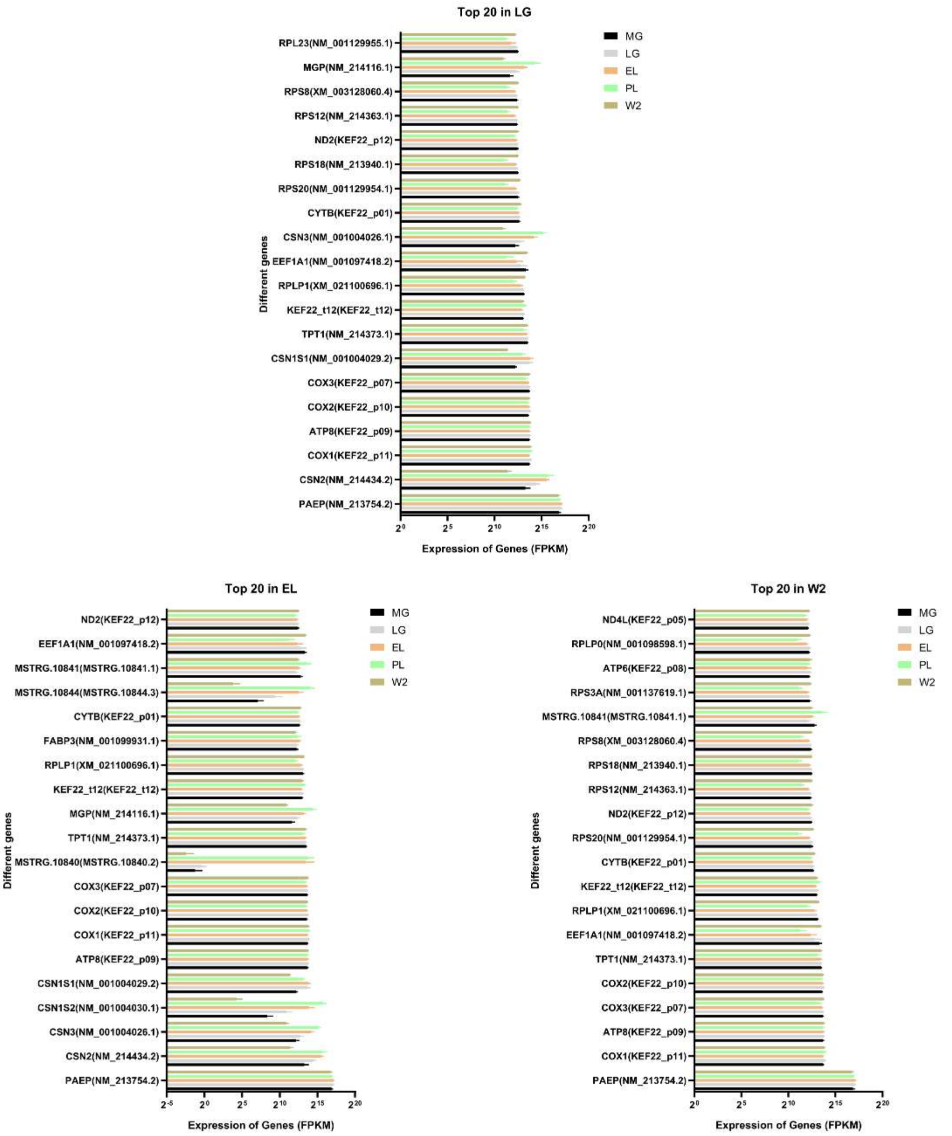

Supplement 7. Expression profiles of the top 20 highly expressed genes at different mammary gland developmental stages. Bar plots show the expression levels (FPKM) of the top 20 highly expressed genes selected from late gestation (LG), early lactation (EL), and early involution (W2) stages. The expression of these genes is compared across five stages: mid-gestation (MG), late gestation (LG), early lactation (EL), peak lactation (PL), and early involution (W2).
