# Supplementary material for: Time-resolved transcriptomic profiling of mammary gland tissue during ductal morphogenesis, lactation activation, and involution in sows
Source: Anim Biosci. 2025 Nov 14;39(5):250560. doi: 10.5713/ab.250560 (PMC13175048; doi:10.5713/ab.250560)
Supplement: Supplementary file 8 [file ab-250560-Supplement-8.pdf]

Supplement 8. Differentially expressed transcrips identified across different developmental stages of the mammary gland. Due to the large file size, the complete raw count matrix has been deposited in Figshare and is publicly available at https://doi.org/10.6084/m9.figshare.31015387.
