# Supplementary material for: Time-resolved transcriptomic profiling of mammary gland tissue during ductal morphogenesis, lactation activation, and involution in sows
Source: Anim Biosci. 2025 Nov 14;39(5):250560. doi: 10.5713/ab.250560 (PMC13175048; doi:10.5713/ab.250560)
Supplement: Supplementary file 9 [file ab-250560-Supplement-9.pdf]

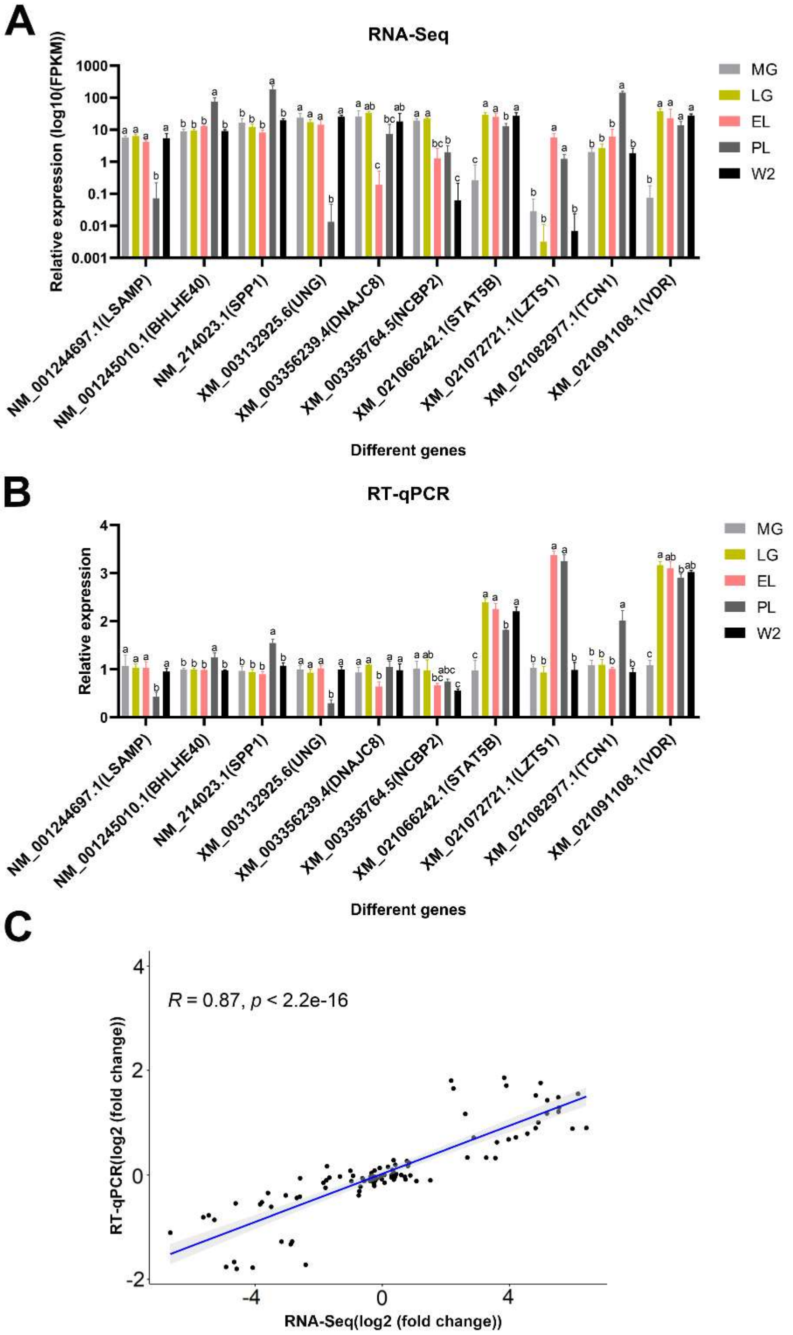

Supplement 9. Validation of RNA-Seq results by RT-qPCR analysis. (A) Relative expression levels (log10-transformed FPKM) of selected differentially expressed genes detected by RNA-Seq across five mammary gland developmental stages: mid-gestation (MG), late gestation (LG), early lactation (EL), peak lactation (PL), and early involution (W2). Different letters above the bars indicate significant differences ($p < 0.05$). (B) Relative expression levels of the same genes validated by RT-qPCR. (C) Correlation analysis between RNA-Seq and RT-qPCR results. A strong positive correlation was observed ($R = 0.87$, $p < 2.2e\text{-}16$), supporting the reliability of the RNA-Seq data.
