# Supplementary material for: Time-resolved transcriptomic profiling of mammary gland tissue during ductal morphogenesis, lactation activation, and involution in sows
Source: Anim Biosci. 2025 Nov 14;39(5):250560. doi: 10.5713/ab.250560 (PMC13175048; doi:10.5713/ab.250560)
Supplement: Supplementary file 11 [file ab-250560-Supplement-11.pdf]

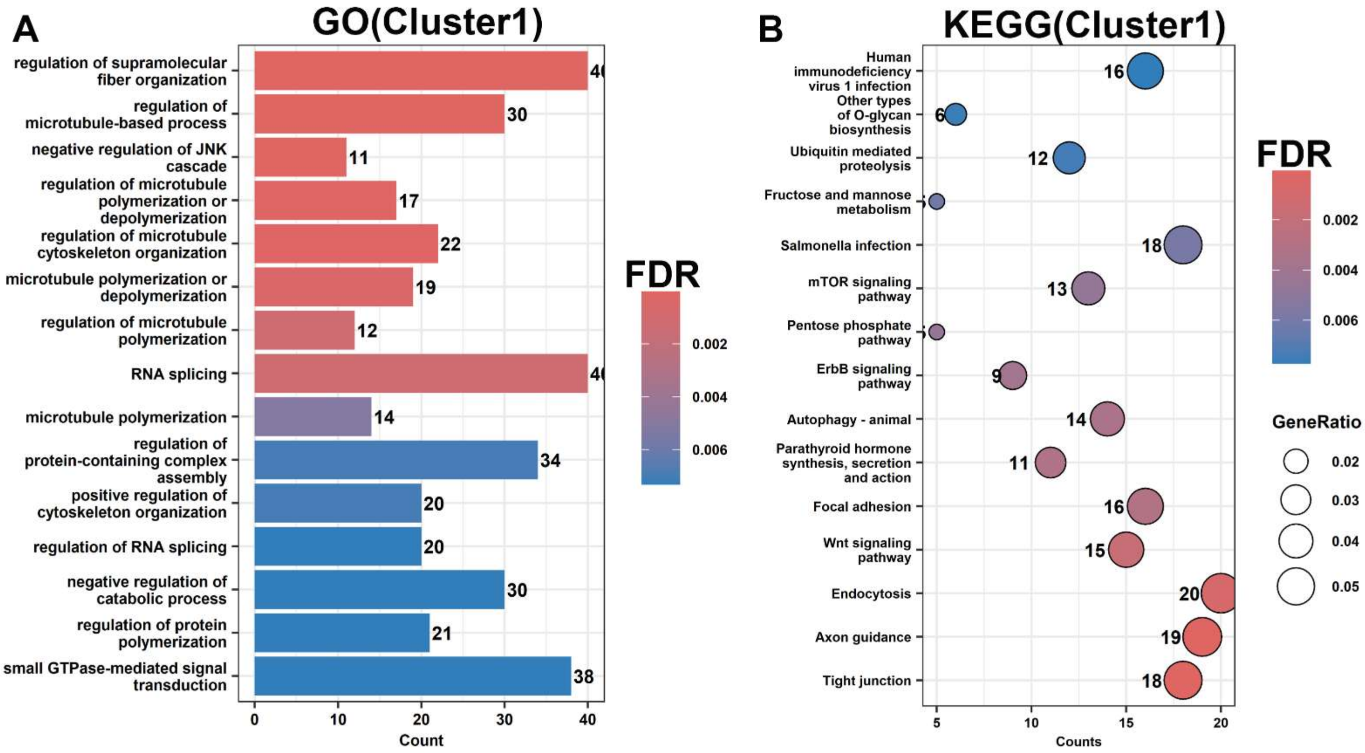

Supplement 11. Functional enrichment analysis of genes in Cluster 1. (A) Gene Ontology (GO) enrichment analysis for genes in Cluster 1. Top enriched biological processes included regulation of supramolecular fiber organization, microtubule-based processes, and RNA splicing. The bar color represents the false discovery rate (FDR), and the number at the end of each bar indicates the gene count. (B) Kyoto Encyclopedia of Genes and Genomes (KEGG) pathway enrichment analysis for Cluster 1 genes. The size of each bubble represents the gene ratio, and the color gradient represents the FDR value. Enriched pathways included tight junction, axon guidance, endocytosis, and Wnt signaling pathway.
