# Supplementary material for: Time-resolved transcriptomic profiling of mammary gland tissue during ductal morphogenesis, lactation activation, and involution in sows
Source: Anim Biosci. 2025 Nov 14;39(5):250560. doi: 10.5713/ab.250560 (PMC13175048; doi:10.5713/ab.250560)
Supplement: Supplementary file 12 [file ab-250560-Supplement-12.pdf]

Supplement 12. GO and KEGG functional enrichment analysis for each cluster of differentially expressed transcripts. Due to the large file size, the complete raw count matrix has been deposited in Figshare and is publicly available at https://doi.org/10.6084/m9.figshare.31015387.
