# Supplementary material for: Time-resolved transcriptomic profiling of mammary gland tissue during ductal morphogenesis, lactation activation, and involution in sows
Source: Anim Biosci. 2025 Nov 14;39(5):250560. doi: 10.5713/ab.250560 (PMC13175048; doi:10.5713/ab.250560)
Supplement: Supplementary file 14 [file ab-250560-Supplement-14.pdf]

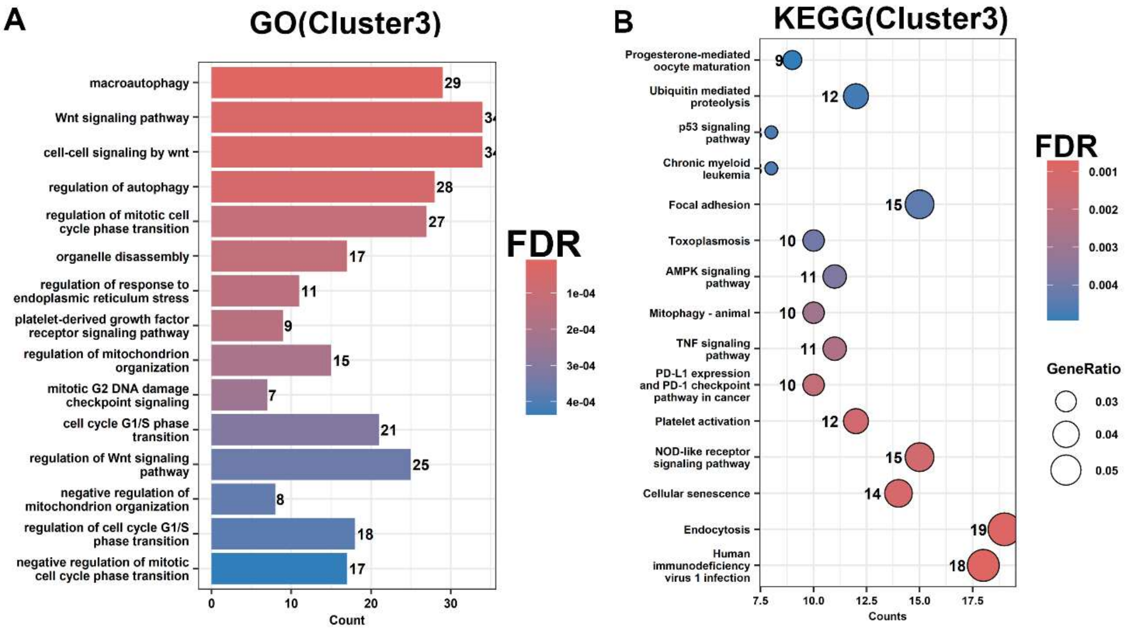

Supplement 14. Functional enrichment analysis of genes in Cluster 3. (A) Gene Ontology (GO) enrichment analysis for genes in Cluster 3. Top enriched biological processes included macroautophagy, Wnt signaling pathway, cell-cell signaling by Wnt, and regulation of autophagy. The bar color represents the false discovery rate (FDR), and the number at the end of each bar indicates the gene count. (B) Kyoto Encyclopedia of Genes and Genomes (KEGG) pathway enrichment analysis for Cluster 3 genes. The size of each bubble represents the gene ratio, and the color gradient represents the FDR value. Enriched pathways included endocytosis, cellular senescence, PD-L1 expression and PD-1 checkpoint pathway in cancer, and AMPK signaling pathway.
