# Supplementary material for: Time-resolved transcriptomic profiling of mammary gland tissue during ductal morphogenesis, lactation activation, and involution in sows
Source: Anim Biosci. 2025 Nov 14;39(5):250560. doi: 10.5713/ab.250560 (PMC13175048; doi:10.5713/ab.250560)
Supplement: Supplementary file 16 [file ab-250560-Supplement-16.pdf]

Supplement 16. WGCNA results and correlation analysis between gene co-expression modules and the five developmental stages. Due to the large file size, the complete raw count matrix has been deposited in Figshare and is publicly available at https://doi.org/10.6084/m9.figshare.31015387.
