# Supplementary material for: Time-resolved transcriptomic profiling of mammary gland tissue during ductal morphogenesis, lactation activation, and involution in sows
Source: Anim Biosci. 2025 Nov 14;39(5):250560. doi: 10.5713/ab.250560 (PMC13175048; doi:10.5713/ab.250560)
Supplement: Supplementary file 17 [file ab-250560-Supplement-17.pdf]

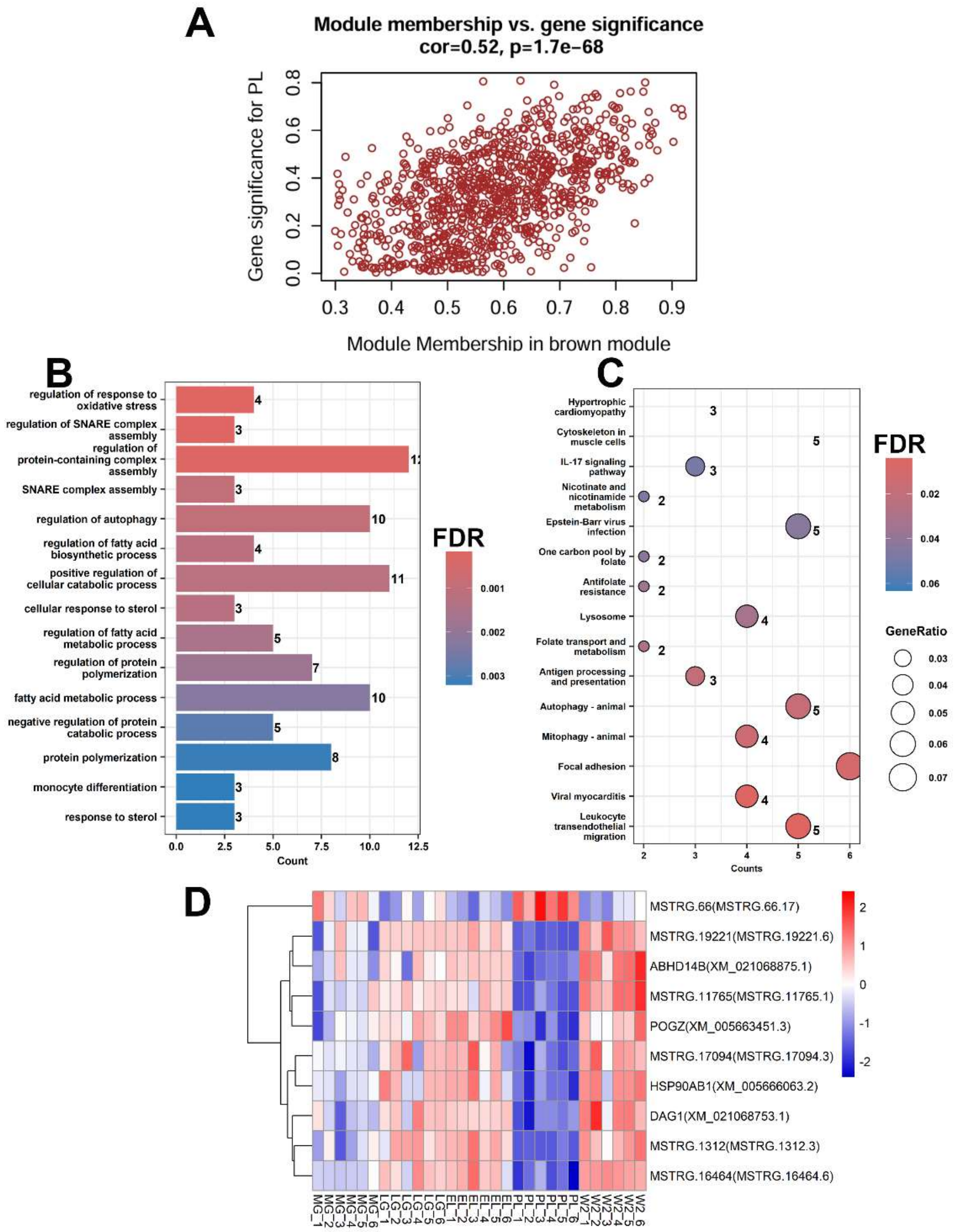

Supplement 17. WGCNA analysis and functional annotation of the brown module. (A) Correlation between gene significance for peak lactation (PL) and module membership in the brown module. A positive correlation was observed (cor = 0.52, p = 1.7e-68). (B) Gene Ontology (GO) enrichment analysis of genes in the brown module. The bar color represents the false discovery rate (FDR), and the number at the end of each bar indicates the gene count. (C) Kyoto Encyclopedia of Genes and Genomes (KEGG) pathway enrichment analysis for genes in the brown module. The size of each bubble represents the gene ratio, and the color gradient represents the FDR value. (D) Heatmap showing the expression patterns of the top 10 hub genes in the brown module across different samples.
