# Supplementary material for: Time-resolved transcriptomic profiling of mammary gland tissue during ductal morphogenesis, lactation activation, and involution in sows
Source: Anim Biosci. 2025 Nov 14;39(5):250560. doi: 10.5713/ab.250560 (PMC13175048; doi:10.5713/ab.250560)
Supplement: Supplementary file 24 [file ab-250560-Supplement-24.pdf]

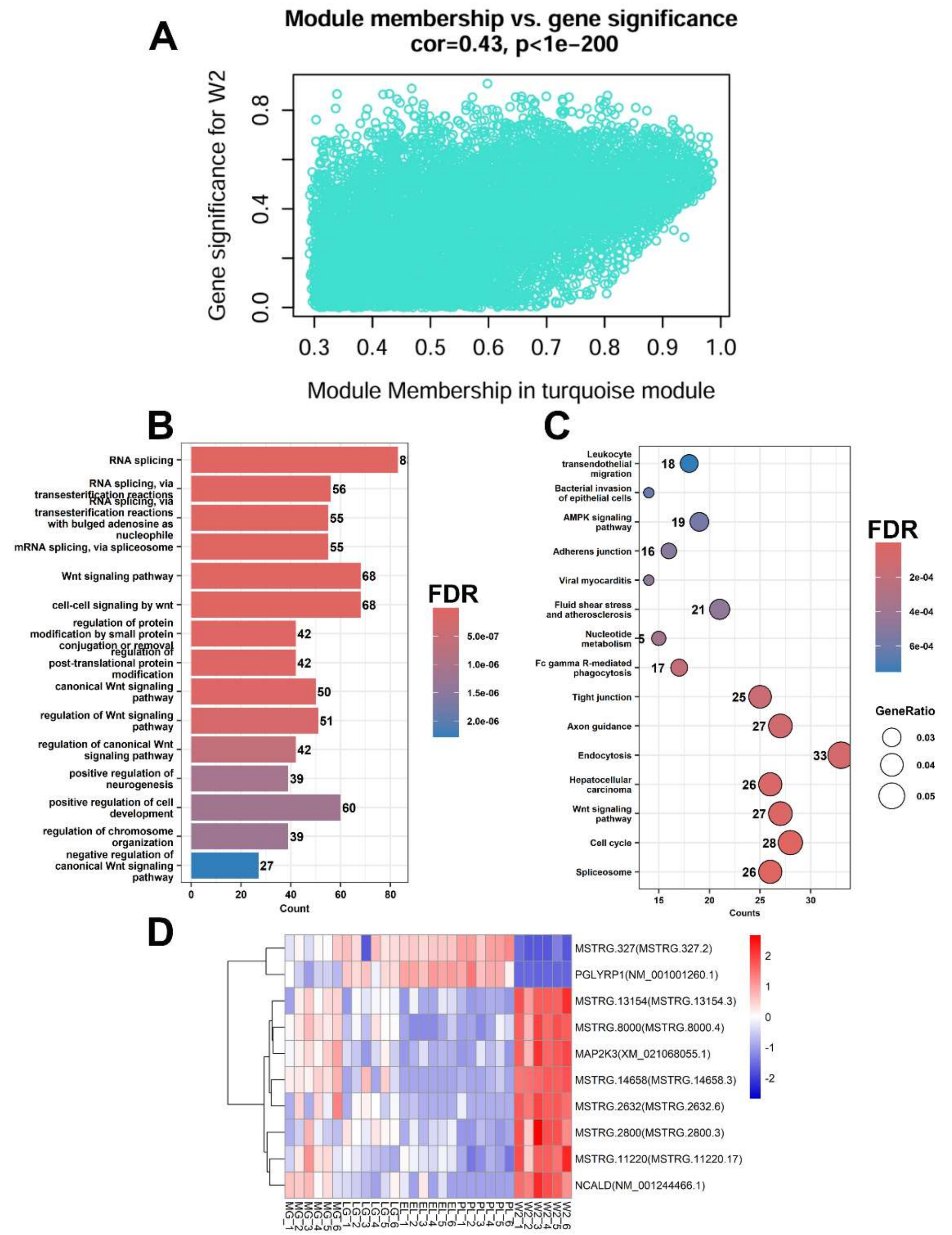

Supplement 24. Analysis of the turquoise module highly associated with W2 stage. (A) Scatter plot of module membership versus gene significance for W2 stage in the turquoise module. A positive correlation was observed (cor = 0.43, $p < 1e\text{-}200$), indicating that genes with higher module membership tend to have higher biological relevance to W2. (B) Bar plot showing GO biological process enrichment analysis for genes in the turquoise module. The color gradient indicates the FDR value, and the number at the right of each bar represents the gene count in each GO term. (C) Bubble plot showing KEGG pathway enrichment analysis for genes in the turquoise module. Bubble size represents the GeneRatio, and color represents the FDR value. (D) Heatmap showing the expression patterns of top hub genes in the turquoise module across different samples. Red indicates high expression and blue indicates low expression.
