# Supplementary material for: Time-resolved transcriptomic profiling of mammary gland tissue during ductal morphogenesis, lactation activation, and involution in sows
Source: Anim Biosci. 2025 Nov 14;39(5):250560. doi: 10.5713/ab.250560 (PMC13175048; doi:10.5713/ab.250560)
Supplement: Supplementary file 25 [file ab-250560-Supplement-25.pdf]

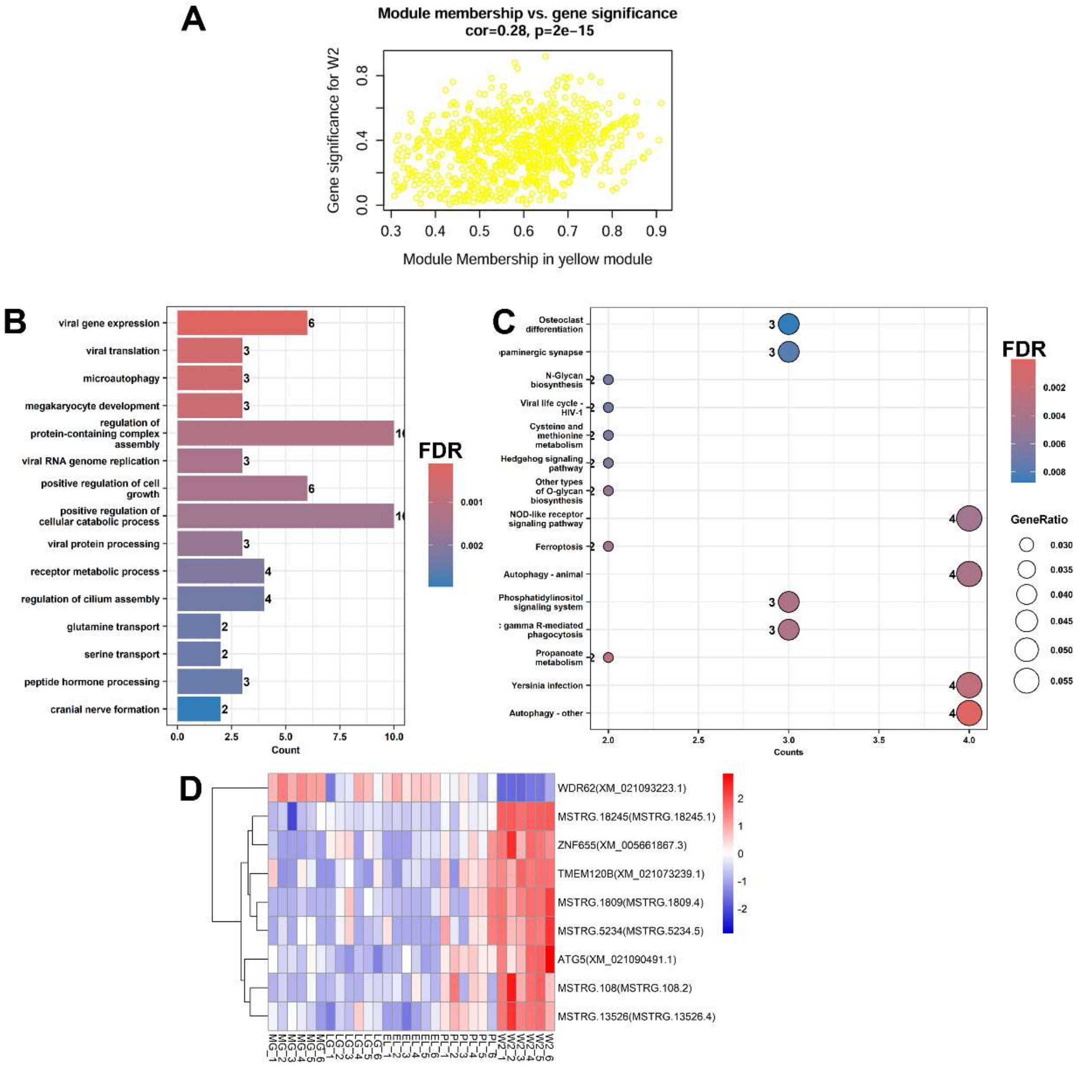

Supplement 25. Analysis of the yellow module associated with W2 stage. (A) Scatter plot of module membership versus gene significance for W2 stage in the yellow module. A moderate positive correlation was observed (cor = 0.28, p = 2e-15), suggesting genes with higher module membership are moderately related to W2 biological traits. (B) Bar plot showing GO biological process enrichment analysis for genes in the yellow module. The color gradient represents the FDR value, and the number at the right of each bar indicates the gene count within each GO term. (C) Bubble plot showing KEGG pathway enrichment analysis for genes in the yellow module. Bubble size indicates the GeneRatio, and color represents the FDR value. (D) Heatmap showing the expression patterns of top hub genes in the yellow module across different samples. Red indicates high expression and blue indicates low expression.
