# Supplementary material for: Time-resolved transcriptomic profiling of mammary gland tissue during ductal morphogenesis, lactation activation, and involution in sows
Source: Anim Biosci. 2025 Nov 14;39(5):250560. doi: 10.5713/ab.250560 (PMC13175048; doi:10.5713/ab.250560)
Supplement: Supplementary file 26 [file ab-250560-Supplement-26.pdf]

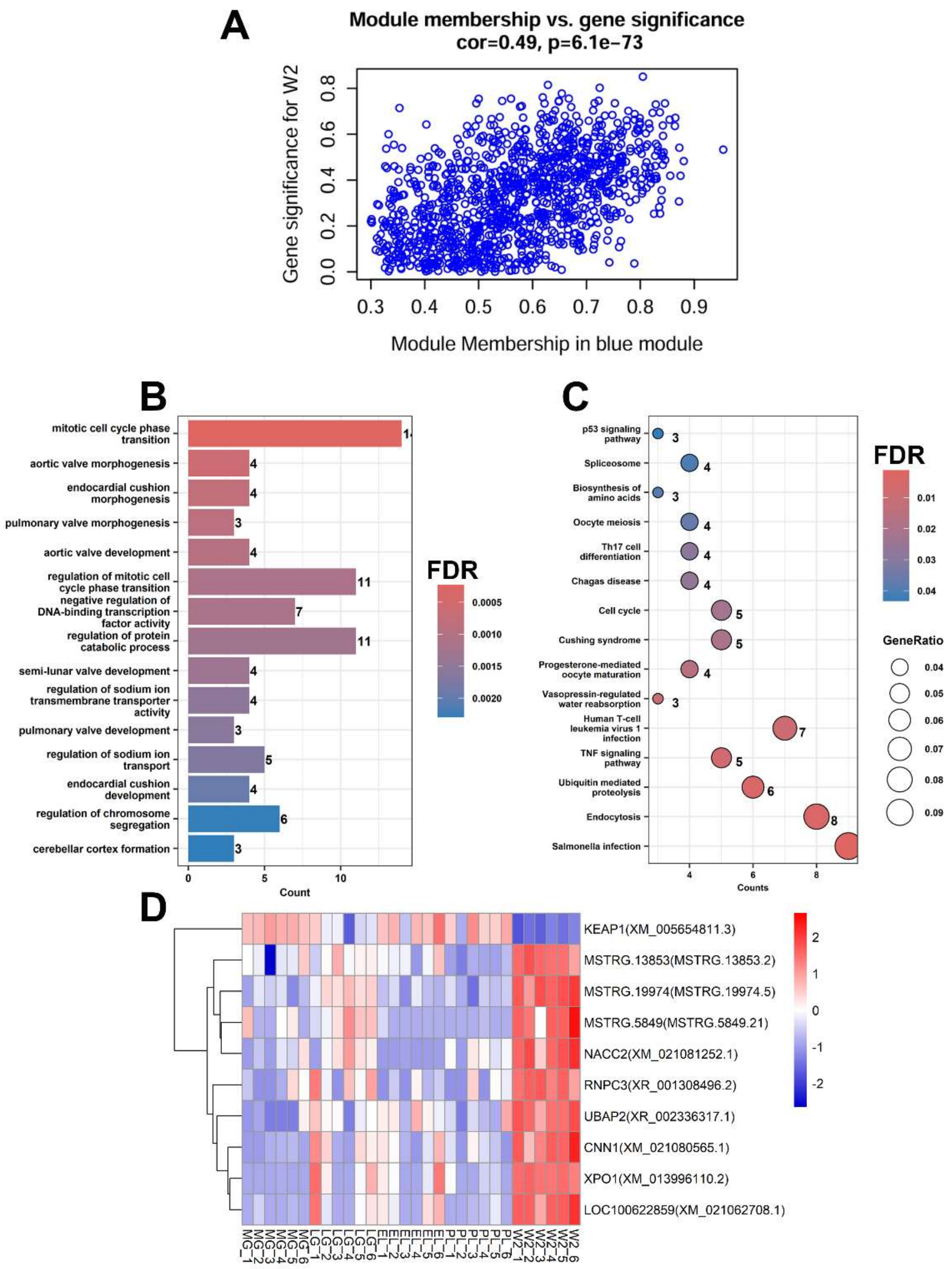

Supplement 26. Analysis of the blue module associated with W2 stage. (A) Scatter plot of module membership versus gene significance for W2 stage in the blue module. A strong positive correlation was observed (cor = 0.49, p = 6.1e-73), suggesting that genes with higher module membership are highly relevant to W2 biological traits. (B) Bar plot showing GO biological process enrichment analysis for genes in the blue module. The color gradient represents the FDR value, and the number at the right of each bar indicates the gene count within each GO term. (C) Bubble plot showing KEGG pathway enrichment analysis for genes in the blue module. Bubble size indicates the GeneRatio, and color represents the FDR value. (D) Heatmap showing the expression patterns of top hub genes in the blue module across different samples. Red indicates high expression and blue indicates low expression.
