# Supplementary material for: Time-resolved transcriptomic profiling of mammary gland tissue during ductal morphogenesis, lactation activation, and involution in sows
Source: Anim Biosci. 2025 Nov 14;39(5):250560. doi: 10.5713/ab.250560 (PMC13175048; doi:10.5713/ab.250560)
Supplement: Supplementary file 27 [file ab-250560-Supplement-27.pdf]

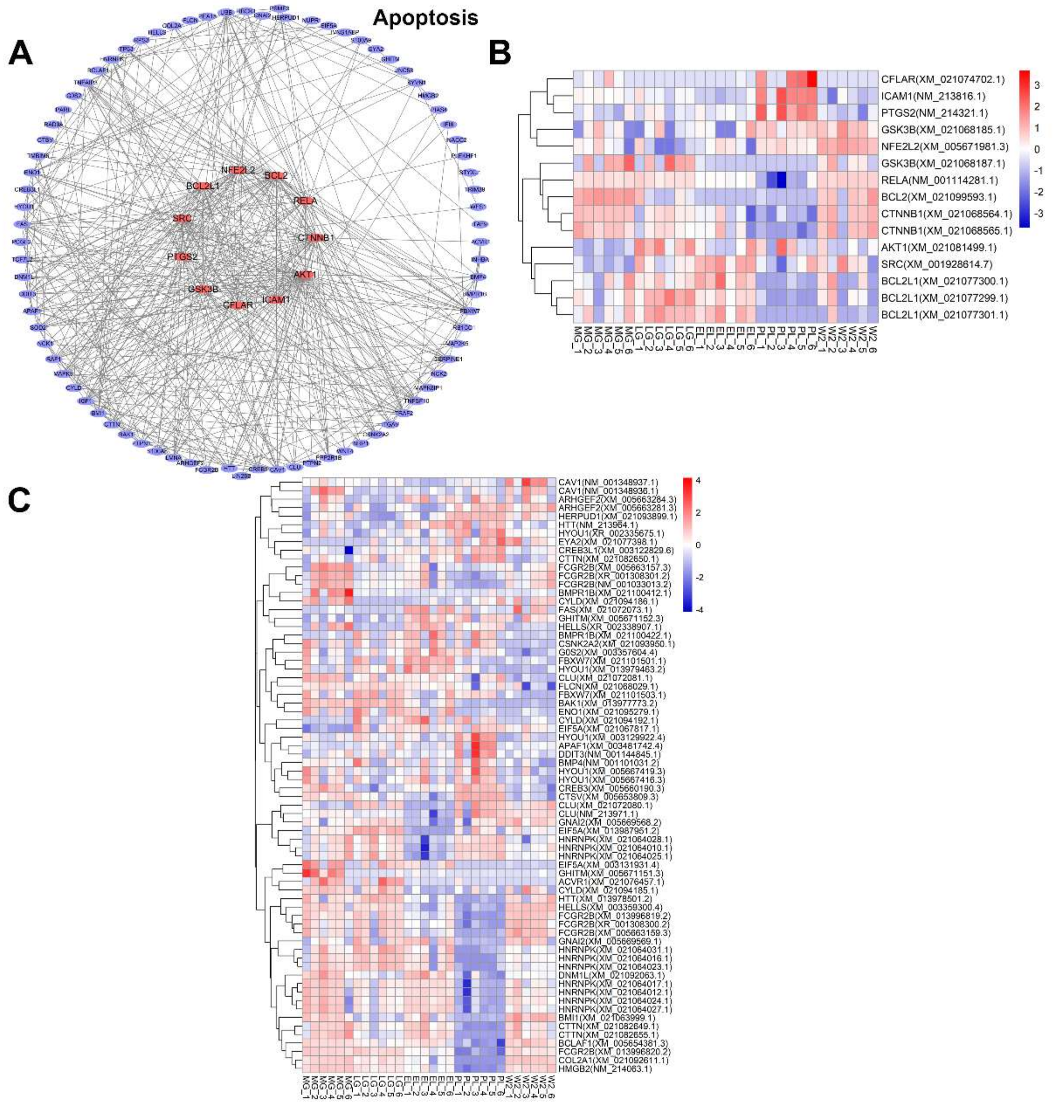

Supplement 27. Construction and expression analysis of the apoptosis-related core regulatory network. (A) Network diagram of the apoptosis-related genes in mammary gland. Red nodes represent key hub genes, and blue nodes represent other apoptosis-associated genes. Edges indicate potential regulatory interactions among genes. (B) Heatmap showing the expression profiles of core hub genes in the apoptosis network across different samples. Red indicates high expression and blue indicates low expression. (C) Heatmap showing the expression patterns of additional apoptosis-related genes across samples. Red indicates high expression and blue indicates low expression.
