# Supplementary material for: Time-resolved transcriptomic profiling of mammary gland tissue during ductal morphogenesis, lactation activation, and involution in sows
Source: Anim Biosci. 2025 Nov 14;39(5):250560. doi: 10.5713/ab.250560 (PMC13175048; doi:10.5713/ab.250560)
Supplement: Supplementary file 29 [file ab-250560-Supplement-29.pdf]

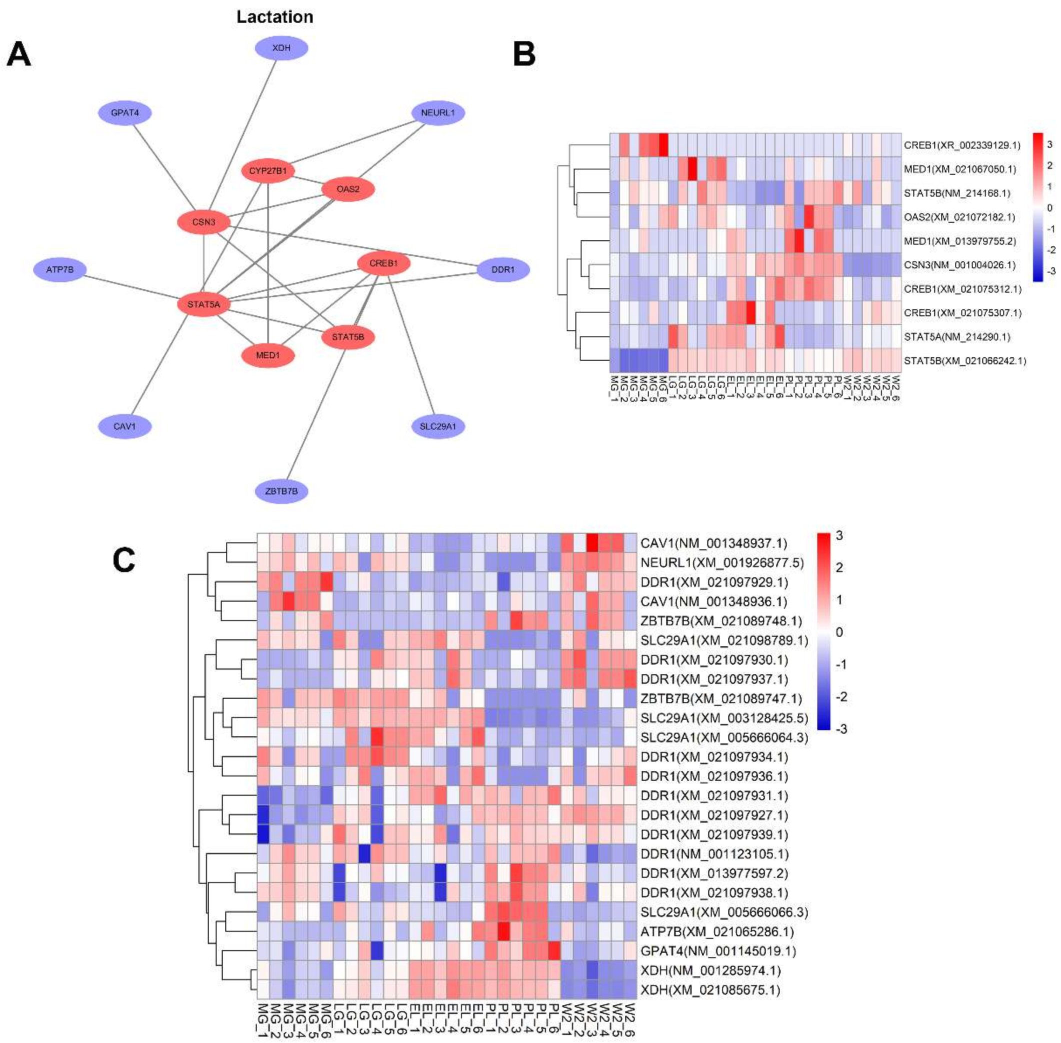

Supplement 29. Construction and expression analysis of the lactation-related gene network. (A) Network diagram of lactation-related genes. Red nodes represent key regulatory genes, and blue nodes represent other lactation-associated genes. Edges indicate potential regulatory relationships among genes. (B) Heatmap showing the expression profiles of selected key lactation-related genes across different samples. Red indicates high expression and blue indicates low expression. (C) Heatmap showing the expression patterns of additional lactation-associated genes across samples. Red indicates high expression and blue indicates low expression.
