# Supplementary material for: Time-resolved transcriptomic profiling of mammary gland tissue during ductal morphogenesis, lactation activation, and involution in sows
Source: Anim Biosci. 2025 Nov 14;39(5):250560. doi: 10.5713/ab.250560 (PMC13175048; doi:10.5713/ab.250560)
Supplement: Supplementary file 30 [file ab-250560-Supplement-30.pdf]

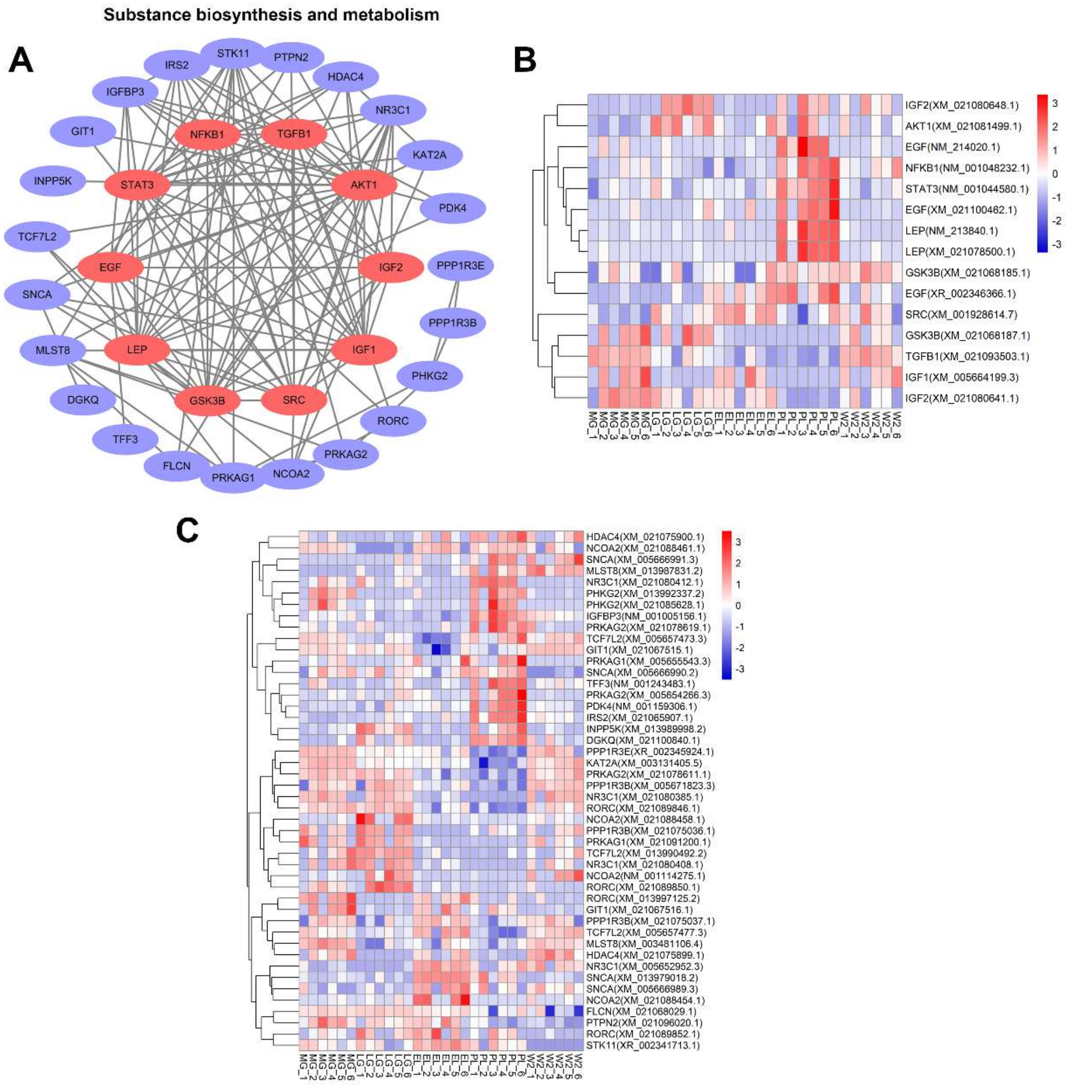

Supplement 30. Construction and expression analysis of the substance biosynthesis and metabolism-related gene network. (A) Network diagram of substance biosynthesis and metabolism-related genes. Red nodes represent key regulatory genes, and blue nodes represent other associated genes. Edges indicate potential regulatory relationships among genes. (B) Heatmap showing the expression profiles of selected key biosynthesis and metabolism-related genes across different samples. Red indicates high expression and blue indicates low expression. (C) Heatmap showing the expression patterns of additional biosynthesis and metabolism-associated genes across samples. Red indicates high expression and blue indicates low expression.
