# Supplementary material for: Time-resolved transcriptomic profiling of mammary gland tissue during ductal morphogenesis, lactation activation, and involution in sows
Source: Anim Biosci. 2025 Nov 14;39(5):250560. doi: 10.5713/ab.250560 (PMC13175048; doi:10.5713/ab.250560)
Supplement: Supplementary file 21 [file ab-250560-Supplement-21.pdf]

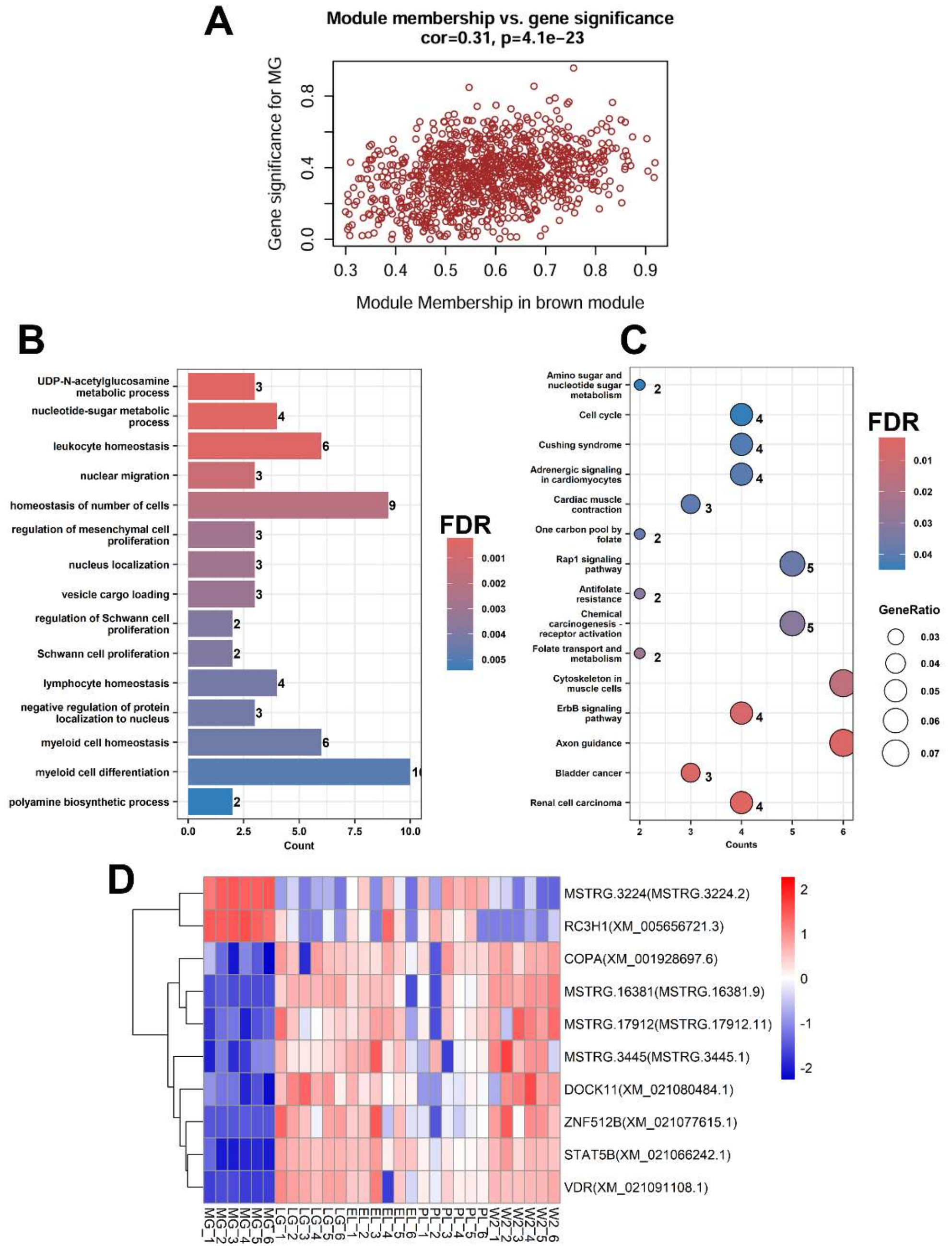

Supplement 21. Correlation analysis and functional enrichment of the brown module associated with the mid-gestation (MG) stage. (A) Correlation between gene significance (GS) for MG and module membership (MM) in the brown module. The x-axis represents module membership, and the y-axis represents gene significance for MG. The correlation coefficient is 0.31, p = 4.1e-23. (B) GO Biological Process enrichment analysis of genes in the brown module. The x-axis shows the gene count, and color intensity indicates FDR-corrected significance. (C) KEGG pathway enrichment analysis of genes in the brown module. Circle size represents gene ratio, and color indicates FDR-corrected p-value. (D) Heatmap showing the expression patterns of key genes in the brown module. Rows represent genes and columns represent samples, with color indicating normalized expression levels (red for high expression, blue for low expression).
