# Supplementary material for: Time-resolved transcriptomic profiling of mammary gland tissue during ductal morphogenesis, lactation activation, and involution in sows
Source: Anim Biosci. 2025 Nov 14;39(5):250560. doi: 10.5713/ab.250560 (PMC13175048; doi:10.5713/ab.250560)
Supplement: Supplementary file 22 [file ab-250560-Supplement-22.pdf]

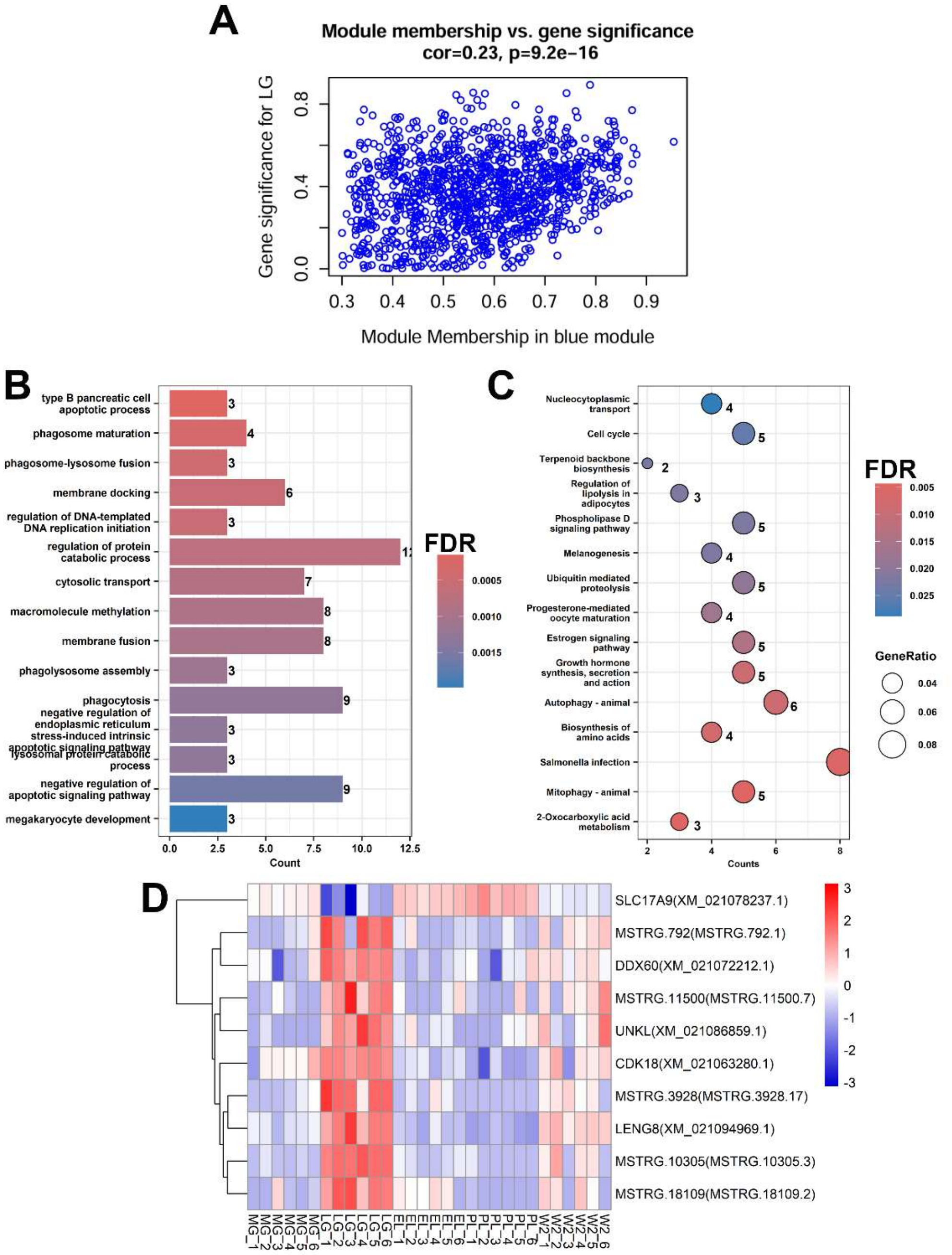

Supplement 22. Correlation analysis and functional enrichment of the blue module associated with the late gestation (LG) stage. (A) Correlation between gene significance (GS) for LG and module membership (MM) in the blue module. The x-axis represents module membership, and the y-axis represents gene significance for LG. The correlation coefficient is 0.23, p = 9.2e-16. (B) GO Biological Process enrichment analysis of genes in the blue module. The x-axis shows the gene count, and color intensity indicates FDR-corrected significance. (C) KEGG pathway enrichment analysis of genes in the blue module. Circle size represents gene ratio, and color indicates FDR-corrected p-value. (D) Heatmap showing the expression patterns of key genes in the blue module. Rows represent genes and columns represent samples, with color indicating normalized expression levels (red for high expression, blue for low expression).
