# Supplementary material for: Time-resolved transcriptomic profiling of mammary gland tissue during ductal morphogenesis, lactation activation, and involution in sows
Source: Anim Biosci. 2025 Nov 14;39(5):250560. doi: 10.5713/ab.250560 (PMC13175048; doi:10.5713/ab.250560)
Supplement: Supplementary file 23 [file ab-250560-Supplement-23.pdf]

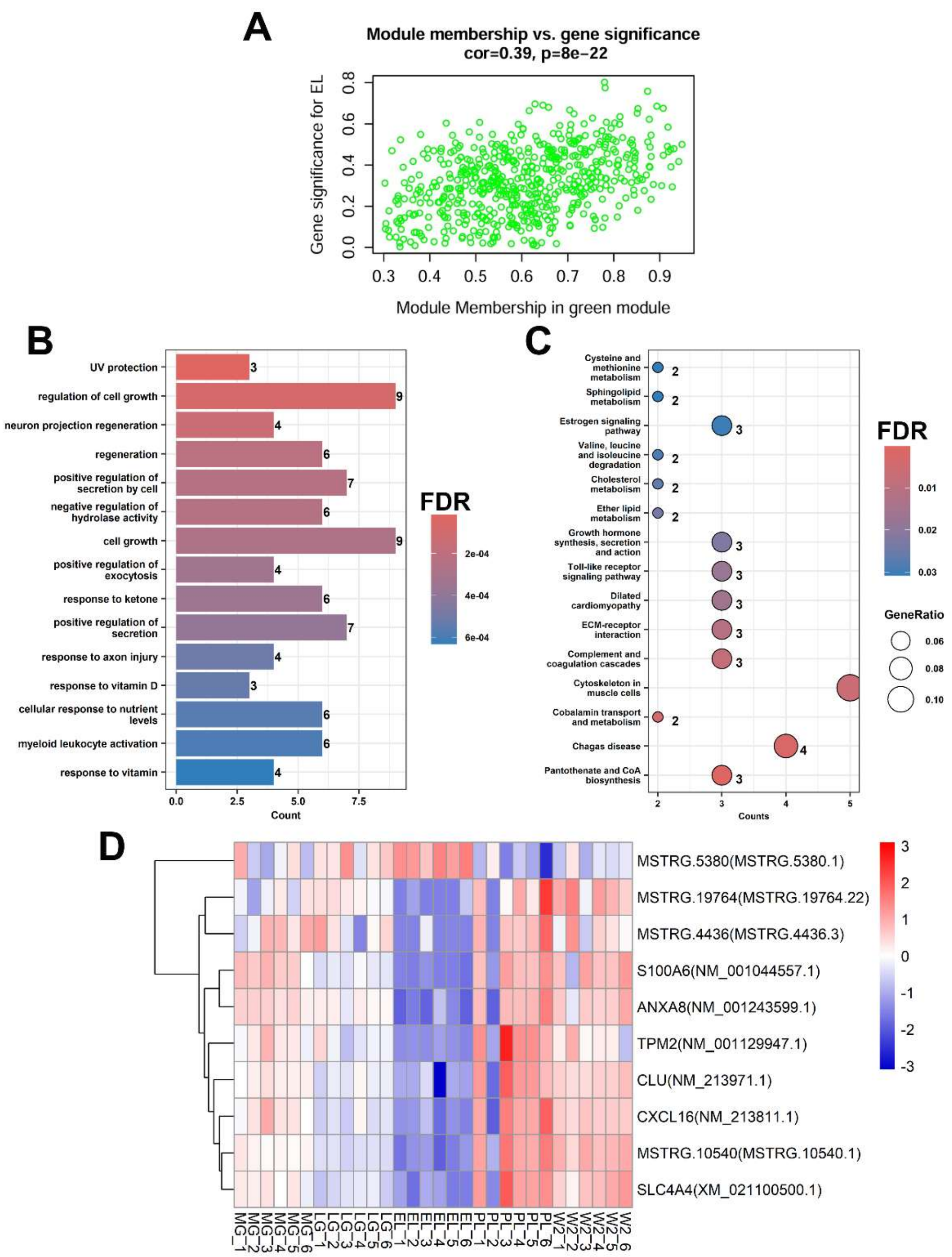

Supplement 23. Correlation analysis and functional enrichment of the green module associated with the early lactation (EL) stage. (A) Correlation between gene significance (GS) for EL and module membership (MM) in the green module. The x-axis represents module membership, and the y-axis represents gene significance for EL. The correlation coefficient is 0.39, p = 8e-22. (B) GO Biological Process enrichment analysis of genes in the green module. The x-axis shows the gene count, and color intensity indicates FDR-corrected significance. (C) KEGG pathway enrichment analysis of genes in the green module. Circle size represents gene ratio, and color indicates FDR-corrected p-value. (D) Heatmap showing the expression patterns of key genes in the green module. Rows represent genes and columns represent samples, with color indicating normalized expression levels (red for high expression, blue for low expression).
