# Supplementary material for: Time-resolved transcriptomic profiling of mammary gland tissue during ductal morphogenesis, lactation activation, and involution in sows
Source: Anim Biosci. 2025 Nov 14;39(5):250560. doi: 10.5713/ab.250560 (PMC13175048; doi:10.5713/ab.250560)
Supplement: Supplementary file 28 [file ab-250560-Supplement-28.pdf]

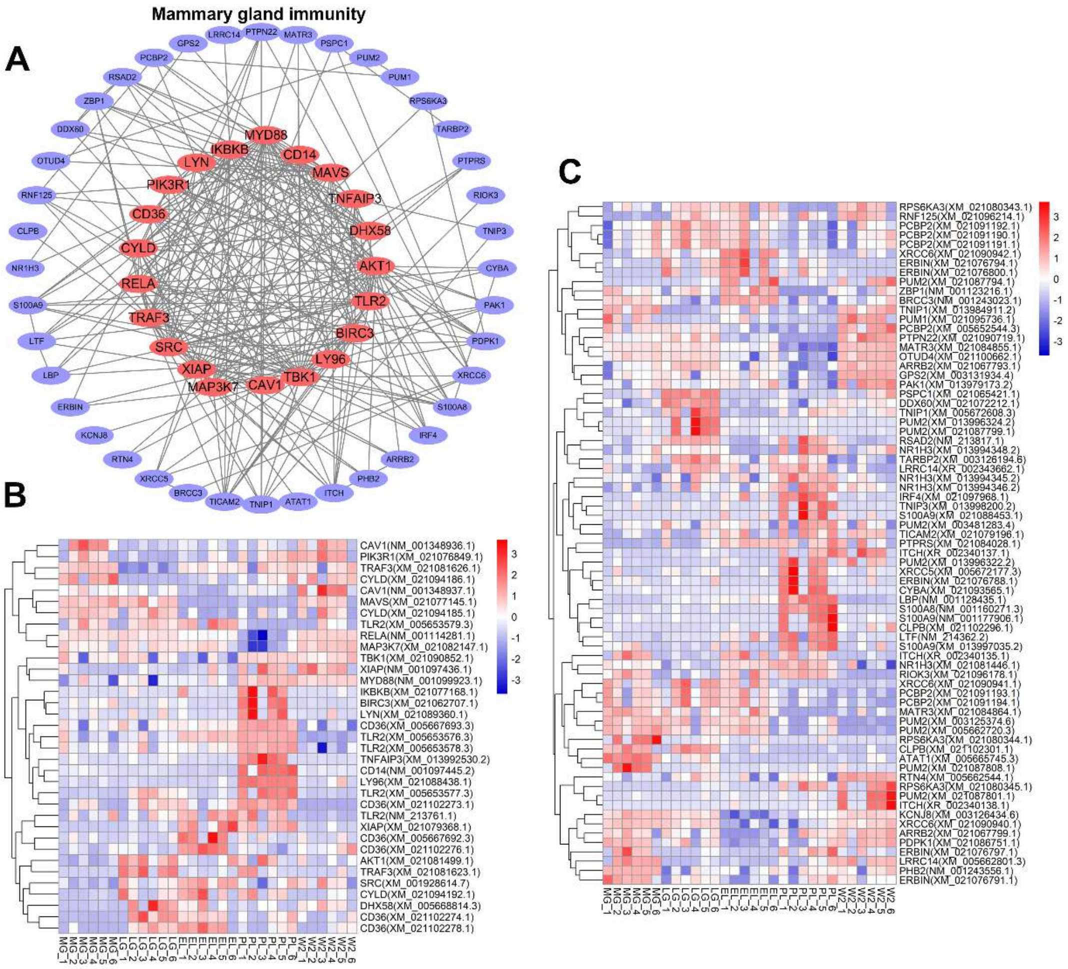

Supplement 28. Construction and expression analysis of the mammary gland immunity-related gene network. (A) Network diagram of mammary gland immunity-related genes. Red nodes represent key regulatory genes, and blue nodes represent other immunity-associated genes. Edges indicate potential interactions among genes. (B) Heatmap showing the expression profiles of selected key immunity-related genes across different samples. Red indicates high expression and blue indicates low expression. (C) Heatmap showing the expression patterns of additional immunity-related genes across samples. Red indicates high expression and blue indicates low expression.
